# Identification of novel furo[2,3-*d*]pyrimidine based chalcones as potent anti-breast cancer agents: synthesis, *in vitro* and *in vivo* biological evaluation†

**DOI:** 10.1039/d2ra00889k

**Published:** 2022-03-15

**Authors:** Mai A. Mansour, Mamdouh A. Oraby, Zeinab A. Muhammad, Deena S. Lasheen, Hatem M. Gaber, Khaled A. M. Abouzid

**Affiliations:** Pharmaceutical Chemistry Department, Faculty of Pharmacy, Badr University in Cairo Cairo Egypt maiali92@yahoo.com; Pharmacology Department, Faculty of Pharmacy, Badr University in Cairo Cairo Egypt; National Organization for Drug Control and Research Cairo Egypt; Pharmaceutical Chemistry Department, Faculty of Pharmacy, Ain Shams University, Abbassia Cairo 11566 Egypt khaled.abouzid@pharma.asu.edu.eg; Department of Organic and Medicinal Chemistry, Faculty of Pharmacy, University of Sadat City Menoufia Sadat City Egypt

## Abstract

Various substituted synthetic chalcones demonstrated potent anti-cancer activities. In the current study a series of novel furo[2,3-*d*]pyrimidine based chalcones were synthesized as potential anticancer agents. Among the different substituted derivatives, two of the halogen bearing chalcones, 5d and 5e, demonstrated potent anti-proliferative activity against an NCI 59 cell line, with mean GI_50_ values of 2.41 μM and 1.23 μM, respectively. Moreover, both compounds showed pronounced cytotoxic activity (5d; 1.20 ± 0.21, 5e; 1.90 ± 0.32) against the resistant MCF-7 cell line when compared to doxorubicin; 3.30 ± 0.18. Such outcomes provoked the initiation of an *in vivo* anticancer assessment study, where compound 5e revealed comparable results to doxorubicin.

## Abstract 2

Various substituted synthetic chalcones demonstrated potent anti-cancer activities.

## Graphical abstract

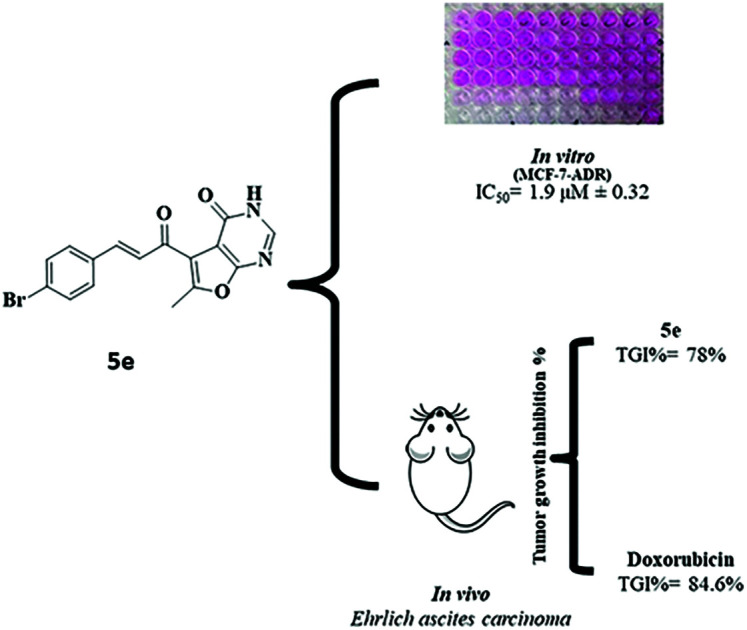

## Introduction

Discovery of a novel anticancer agent with reasonable potency, minor side effects and combating drug resistance has been a continuous vital need in the treatment of the various types of cancer. Chalcones, *i.e.* 1,3-diaryl-2-propen-1-one,^1^ are biosynthetic precursors of flavonoids and are naturally present in many medicinal plants whose bioactivities have been extensively investigated, revealing various biological properties, such as anti-inflammatory,^2^ antibacterial^3^ and antitumor activity.^4^ Initially, their antitumor cytotoxic activity included antiangiogenic activity that induces necrosis and prevents metastasis.^5^ Inhibition of tubulin polymerization, cell cycle disruption, and inhibition of certain kinases required for cell cancer survival as well as apoptosis induction, are other identified antitumor mechanisms.^6^

Apoptosis is regulated through a multi-step pathway, that is demonstrated as cell shrinkage, chromatin condensation, and nuclear and cell fragmentation. These features result in the formation of apoptotic bodies that are then engulfed by neighboring phagocytic cells.^7^ The apoptotic pathway establishes a cascade of events in which activation of caspases takes place. Caspases-3, -6, and -7 are the “executioner” caspases, in which they mediate their effects by the cleavage of specific substrates in the cell.^8^ Particularly, caspase-3 possesses a significant role associated with the dismantling of the cell, rendering its activation crucial in the apoptotic process in mammals.^9^

Even though various chalcone derivatives revealed cytotoxicity against several tumor cell lines, yet, little is known about their mechanism of action and direct molecular targets.^10–12^ Millepachine (compound II), a bioactive natural chalcone, exhibits its antitumor effects in many human cancer cells through inducing obvious G2/M arrest and apoptosis.^13^ Licochalcone C (compound III) is another phenolic chalcone derivative that induces apoptosis in human oral squamous cell carcinoma cells *via* the regulation of the JAK2/STAT3 signalling pathway.^14^

**Fig. 1:**
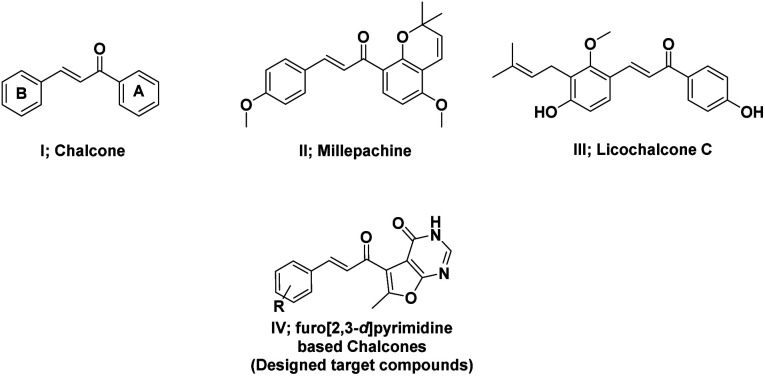
Structures of chalcone, licochalcone A, millepachine, and furopyrimidine based chalcones (designed target compounds).

Numerous heterocyclic chalcones have been synthesized and subjected to biological evaluation as anticancer candidates.^15^ Among these, quinazoline based chalcones presented potent activity against different cell lines.^16^ Moreover, chalcones with halogen substituents in the B-ring have shown improved cytotoxicity against tumor cells when compared to methylated, methoxylated or hydroxylated analogues through apoptosis.^17–19^ Based on the bioisosteric modification strategies, herein, different substituents including halogens were introduced to the novel scaffold, furo[2,3-*d*]pyrimidine based chalcones, to investigate their anti-cancer activity (Fig. 1).

## Results and discussion

### Chemistry

**Scheme 1:**
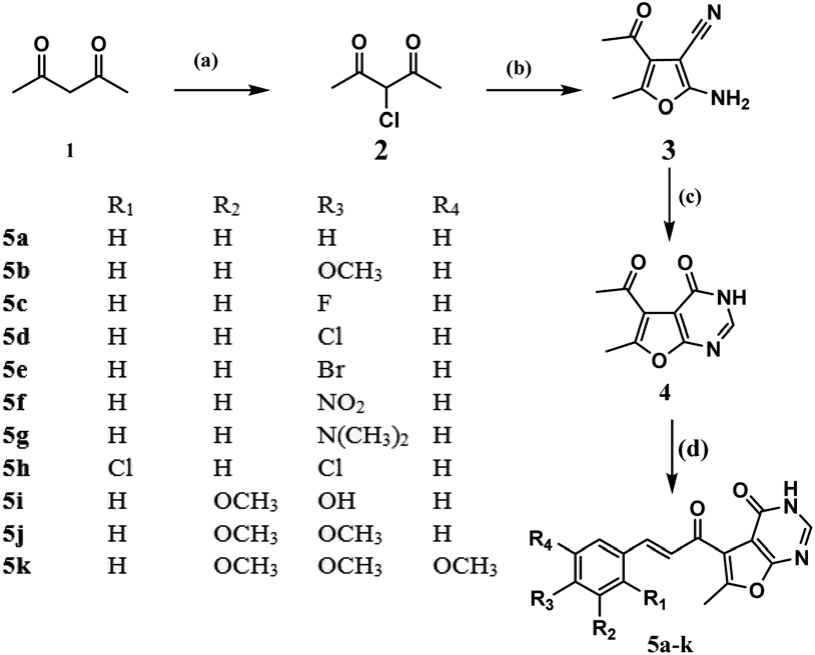
Reagents and conditions: (a) SO_2_Cl_2_, 0 °C; (b) malononitrile, NaOEt, rt, 6 h; (c) HCOOH, acetic anhydride, reflux, 35 h; (d) 60% NaOH, EOH, rt, 5 h, appropriate substituted benzaldehydes.

Synthesis of the designed furo[2,3-*d*]pyrimidine based chalcones started by reacting the active acetyl acetone (1) with sulfuryl chloride to afford the α-chloro acetylacetone (2) as mentioned earlier by Roland Verhié in 1978.^20^ The preparation of the furan derivative (3) was achieved by reacting (2) with malononitrile in a solution of sodium ethoxide according to the reported method.^21^ Thermal cyclocondensation of compound (3) was obtained *via* its reflux with formic acid and acetic anhydride for 35 hours to obtain the furo[2,3-*d*]pyrimidinone derivative (4), to which the appropriate aromatic aldehydes were reacted following the usual Clasien Schmidt condensation to form the corresponding chalcones (5a–k) as presented in Scheme 1: reagents and conditions: (a) SO_2_Cl_2_, 0 °C; (b) malononitrile, NaOEt, rt, 6 h; (c) HCOOH, acetic anhydride, reflux, 35 h; (d) 60% NaOH, EOH, rt, 5 h, appropriate substituted benzaldehydes.

### Biological evaluation

#### *In vitro* anti-proliferative activity against NCI 60-cell line

##### *In vitro* primary single dose (10 μM) full NCI 60 cell panel assay

All the compounds were selected by the National Cancer Institute (NCI), USA for *in vitro* anti-cancer screening. Initially, compounds were screened at the 10 μM dose against NCI 60 cell lines representing nine tumor subpanels, including leukemia, lung, colon, melanoma, renal, prostate, CNS, ovarian, and breast cancer cell lines and showed variable cytotoxic activity with a mean growth% ranging from 5.49% to 102.26%. Best growth inhibition% values were demonstrated within the Leukemia subpanel against the K-562 cell line (54.61, 163.22, 89.21, 42.3, 82.21 and 54.83%) for compounds 5c, 5d, 5e, 5f, 5h and 5k respectively. Growth% accomplished by all compounds is provided (ESI†).

##### *In vitro* five doses NCI 60 cell panel assay

Compounds 5d and 5e showed promising anti-cancer activities against 60 different human cancer cell lines, with mean growth percentage of 26.93% and 5.49%, respectively. Furthermore, they were selected by the NCI for the five-dose assay against 59 NCI cell lines, which gave potent cytotoxic action ranging from (1.09–5.09 μM) and (0.51–4.46 μM), for 5d and 5e respectively (ESI†). Table 1 represents the best GI_50_ values for both compounds 5d and 5e against the nine tumour subpanels.

**Table 1:** GI_50_ values of compounds 5d and 5e against 11 selected NCI cell lines

Panel/cell line	GI50 (μM)	
Cell line	Compound 5d	Compound 5e
**Leukemia**		
K-562	1.82	0.80

**Non-small cell lung cancer**		
HOP-92	1.47	1.35
NCI-H522	1.79	1.89

**Colon cancer**		
HCT-116	1.59	1.26
HCT-15	1.29	1.09

**CNS cancer**		
U251	1.97	1.44

**Melanoma**		
LOX IMVI	1.6	1.03

**Ovarian cancer**		
IGROV1	1.82	1.81

**Renal cancer**		
UO-31	1.09	0.78

**Prostate cancer**		
DU-145	2.82	2.03

**Breast cancer**		
MCF7	1.39	0.51
**Mean GI50**	**2.41**	**1.23**

#### *In vitro* cell cycle analysis by flowcytometry

**Fig. 2:**
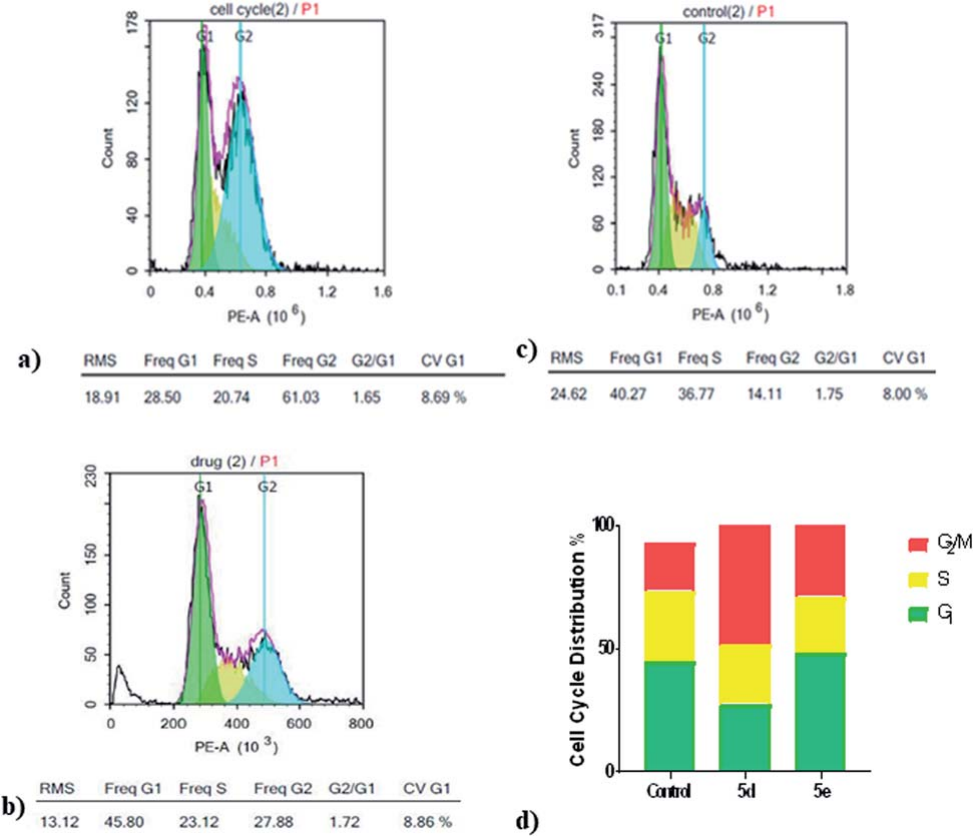
Cell cycle analysis of MCF7 cells after treatment with compounds 5d and 5e for 24 h using flowcytometry. (a) Cell cycle distribution in MCF7 cells after treatment with compound 5d. (b) Cell cycle distribution in MCF7 cells after treatment with compound 5e. (c) Cell cycle distribution in MCF7 cells without treatment. (d) Percentage of MCF7 cells (%) in the G1, S or G2/M phases after treatment with compounds 5d and 5e.

Cell cycle involves of a series of events that result in cell division, DNA replication, and the generation of two daughter cells. Herein, cell cycle development was analyzed using flowcytometry to determine whether the anti-proliferative effect of compounds 5d and 5e, toward MCF7 cells was associated with the induction of cell cycle arrest. Flowcytometry results indicated that compounds 5d and 5e caused an increase in percentage of MCF7 cells lines in the G2/M phase 36.49% and 11.90% at 10 μM, respectively, when compared to the control after 24 h of treatment. Furthermore, percentages of cells in S phase were decreased after treatment with 5d and 5e, while a percentage increase of 3.68% appeared only with compound 5e at the G1 phase and necrotic peak appears as well (Fig. 2). These results indicated that the inhibition of MCF7 cell proliferation by 5d and 5e may be mainly associated with cell cycle arrest in the G2/M phase with an additional necrotic outcome that was achieved by compound 5e.

#### *In vitro* cytotoxic activity against MCF-7-ADR/doxorubicin resistant cancer cell line

Since chalcone based compounds (5d and 5e) showed exceptional anti-proliferative activity against MCF-7 cell line of the breast cancer cell panel (GI_50_5d = 1.39, 5e = 0.51), the wild type, MCF-7-ADR cells were used as a multidrug-resistant breast cancer cell model against the former compounds. Fortunately, the tested compounds showed promising cytotoxic activity against the chosen cell line when compared to doxorubicin as shown in Table 2.

**Table 2:** IC_50_ values of selected target compounds and doxorubicin on MCF-7-ADR cell line

MCF-7-ADR cancer cell line	
Cpd ID	IC_50_ μM
5d	1.2 ± 0.21
5e	1.9 ± 0.32
**Doxorubicin**	3.3 ± 0.18

#### *In vivo* anticancer activity assessment

*In vivo* tumor models are essential for the development of novel therapeutics. Accordingly, compounds (5d, 5e) were selected to be submitted to an *in vivo* efficacy experiment in an established murine Ehrlich ascites carcinoma (EAC) solid tumor model.

EAC cells (5 × 10^5^ cells per 0.1 ml per mouse) were injected subcutaneously into the right hind limb (thigh) of adult swiss albino female mice weighing 15–20 g. Treatment compounds were dissolved in 5% DMSO and injected intraperitoneally at the 5th day (first day of treatment = day 0) after subcutaneous implantation of EAT for 20 days. Compounds (5d, 5e) were tested at the doses of 2.5 and 5 mg kg^−1^, which were orally administered once daily for 20 days.

#### Acute toxicity study

As depicted in Table 3, the first step determination of LD_50_ showed that one mouse was found dead at the dose 1000 mg kg^−1^, whereas no mortality was observed in groups received either 10 or 100 mg kg^−1^ of both 5e and 5d. According to the Lorkes' method, 4 increasing doses (600, 1000, 1600, and 2900 mg kg^−1^; i.p) were injected into 4 different groups (1 mouse each), respectively. Findings of the second step revealed that mice received 600 mg kg^−1^ and 1000 mg kg^−1^ (*D*0; highest dose with no mortality) survived while mice receiving 1600 mg kg^−1^ (*D*100; lowest dose causing mortality) and 2900 mg kg^−1^ died, indicating that both 5e and 5d had a high therapeutic index. Consequently, LD_50_ of both 5e and 5d is equal to the geometric mean of *D*0 and *D*100 when calculated as follows;
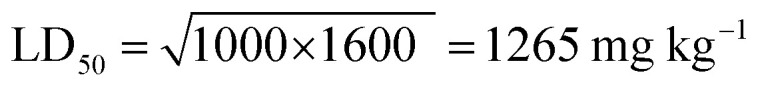


**Table 3:** LD_50_ investigation^a^

	First step investigation		Second step investigation	
	Doses (mg kg^−1^)	Mortality/no. of mice	Dose (mg kg^−1^)	Mortality/no. of mice
5d & 5e	10	0/3	600	0/1
	100	0/3	1000	0/1
	1000	1/3	1600	1/1
			2900	1/1

a
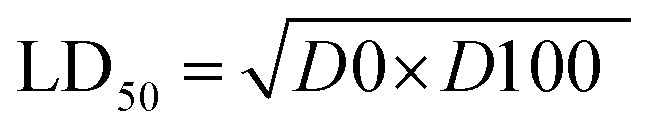
, 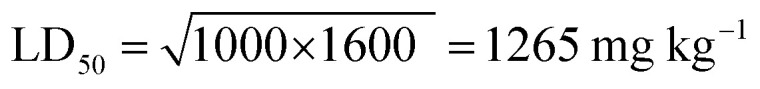.

##### Tumor volume and tumor growth inhibition (TGI) estimation

**Fig. 3:**
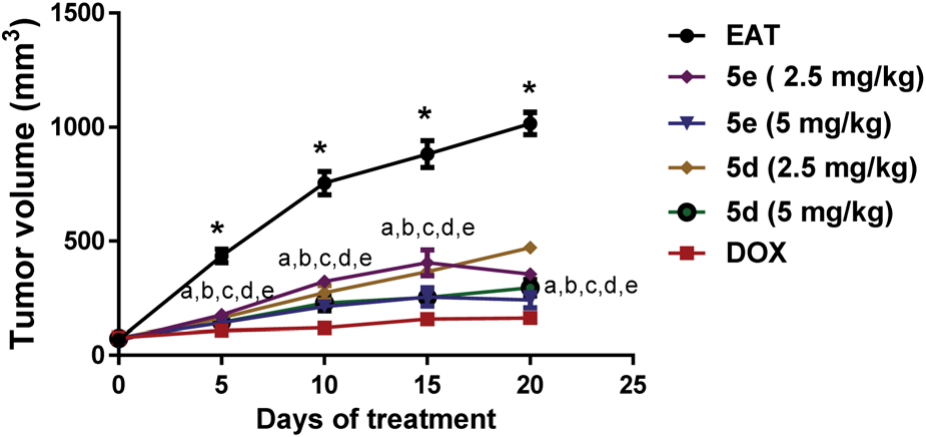
Effect of compounds 5e and 5d on tumor volume. Tested compounds were intraperitoneally injected to EAT mice for 20 days at 2.5 and 5 mg per kg per day. Tumor diameter of each mouse was monitored every 5 days from the first day of treatment (day 0) till day 20 using a digital vernier caliper. Data were presented as mean ± S.E.M of 8 mice for each group, *significant difference *versus* day 0, a–e significant difference of 5e (2.5 mg kg^−1^), 5e (5 mg kg^−1^), 5d (2.5 mg kg^−1^), 5d (5 mg kg^−1^), and doxorubicin, respectively, *versus* the corresponding EAT (statistical analysis was performed by two-way ANOVA, followed by the Tukey's multiple comparison test; at *p* < 0.05).

As represented in Fig. 3: effect of comdpounds 5e and 5d on tumor volume, following intraperitoneal administration of two different doses (2.5 & 5 mg per kg per day) of both tested compounds (5e & 5d) depicted a significant decrease in the tumor volume measured at day 5, 10, 15, and 20 *versus* the corresponding untreated EAT mice. Noticeably, percentage of tumor growth inhibition (TGI%) was 62.4% and 78% for compound 5e at 2.5 & 5 mg kg^−1^ doses, respectively. Likewise, compound 5d at 2.5 & 5 mg kg^−1^ doses markedly decreased tumor volume in EAT mice with TGI% of about 57% and 72.3%, respectively; results that were parallel to DOX group at the higher dose (Table 4).

**Table 4:** Tumor indices and TGI% of EAT control and treated mice. Tested compounds were intraperitoneally injected to EAT mice for 20 days at 2.5 and 5 mg per kg per day. Tumor indices values were presented as mean ± S.E.M of 5 mice for each group. *Significant difference *versus* EAT (statistical analysis was performed by one-way ANOVA, followed by the Tukey's multiple comparison test; at *p* < 0.05). TGI (%); tumor growth inhibition percentage

Parameters/groups	TGI (%)	Tumor index
EAT	0	0.12 ± 0.006
5d (2.5 mg per kg per day)	56.9	0.13 ± 0.002
5d (5 mg per kg per day)	72.3	0.10 ± 0.004
5e (2.5 mg per kg per day)	62.4	0.11 ± 0.007
5e (5 mg per kg per day)	78.0	0.08 ± 0.002*
DOX (2 mg per kg per day)	84.6	0.06 ± 0.008*

For further estimation of the antigrowth activity of both tested compounds, tumor indices were calculated. Neither 5d at its tested doses nor 5e at 2.5 mg per kg per day dose caused marked decline of tumor indices. However, 5 mg per kg per day dose of 5e was able to induce a prominent reduction of tumor index by approximately 35.5% *versus* untreated EAT mice which were comparable to the group treated with doxorubicin (Table 4).

#### Immunoexpression of caspase-3

**Fig. 4:**
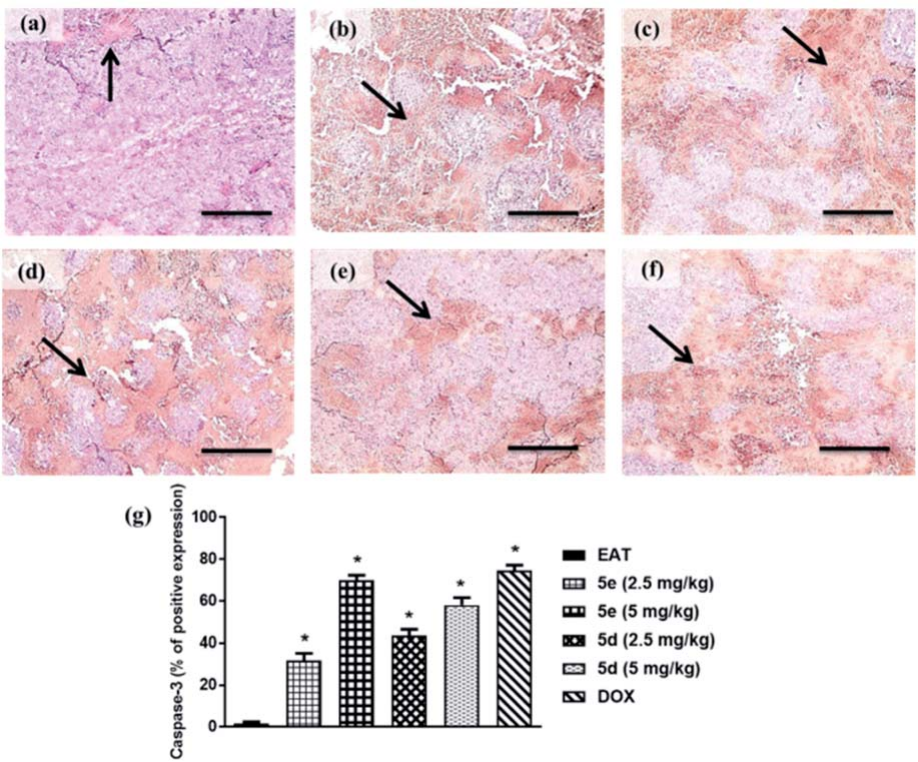
Caspase-3 immune-expression in tumor sections of treated groups (caspase-3 antibody; ×100). (a) EAT, (b) 5e 2.5 mg kg^−1^, (c) 5e 5 mg kg^−1^, (d) 5d 2.5 mg kg^−1^ (d), (e) 5d 5 mg kg^−1^, (f) DOX, (g) % of caspase-3 immunoreactive area to total area through six fields per each section. The black arrow indicates caspase-3 expression within tumor cells. Data were presented as mean ± S.E.M of 5 fields. *Significant difference *versus* EAT (statistical analysis was performed by one-way ANOVA, followed by the Tukey's multiple comparison test; at *p* < 0.05).

Impaired apoptotic machinery is associated with tumor cell growth and development. Among others, Caspase-3 is involved in cell survival and fate by regulating apoptosis.^22^ In this study, a profound surge in caspase-3 immunoreactive area was observed in tumor sections of EAT mice treated with 2.5 or 5 mg kg^−1^ of compounds 5e and 5d by 31.7%, approximately 70%, 43%, and 58%, respectively, relative to untreated ones. Notably, compound 5e exhibited comparable apoptosis to the group treated with doxorubicin as shown in Fig. 4.

#### Oxidative stress biomarkers

**Fig. 5:**
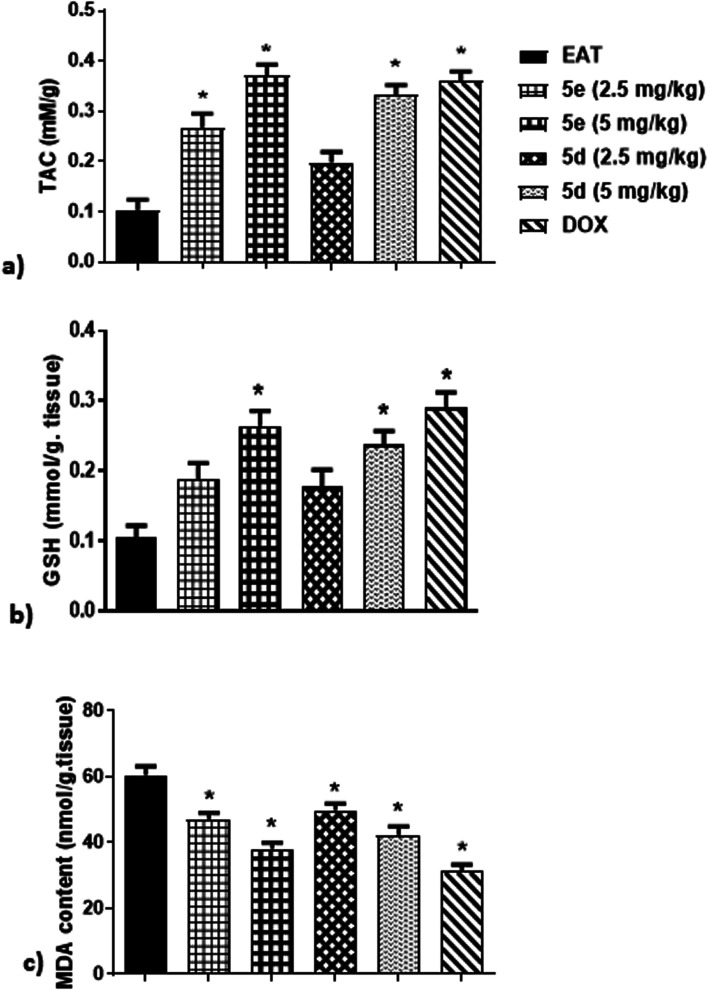
Effect of compounds 5e and 5d on oxidative stress status of EAT mice, (a) TAC, (b) GSH, (c) MDA. Tested compounds were intraperitoneally injected to EAT mice for 20 days at 2.5 and 5 mg per kg per day. Commercially available colorimetric kits were used for determination of TAC, GSH, and MDA. Data were presented as mean ± S.E.M of 6 mice for each group. *Significant difference *versus* EAT (statistical analysis was performed by One-way ANOVA, followed by the Tukey's multiple comparison test; at *p* < 0.05).

It has been suggested that oxidative stress plays a crucial role in tumor propagation, proliferation, and angiogenesis through production of damaged and mutated DNA.^23^ Therefore, this study examined some oxidative stress indices in EAT mice including serum total anti-oxidant capacity (TAC), malondialdehyde (MDA) and glutathione (GSH) levels. As shown in Fig. 5, treatment of EAT mice for 3 weeks with either 5e at 2.5 mg kg^−1^, 5e at 5 mg kg^−1^, or 5d at 5 mg kg^−1^ markedly elevated TAC by 170%, 270%, and 230%, respectively, in comparison to untreated ones. Although test compounds at 2.5 mg kg^−1^ dose failed to attenuate GSH activity in EAT mice, administration of 5 mg per kg per day of 5e and 5d resulted in obvious increase of GSH activity by about 160% and 140%, respectively, related to untreated ones, which were comparable to DOX group (Fig. 5). Contrariwise, intraperitoneal injection of 5e at 2.5 mg kg^−1^, 5e at 5 mg kg^−1^, 5d at 2.5 mg kg^−1^, or 5d at 5 mg kg^−1^ induced an evident reduction of MDA levels in EAT mice by 22%, 37%, 17.5%, and 30%, respectively, as compared to untreated ones (Fig. 5).

#### Histopathological examination

**Fig. 6:**
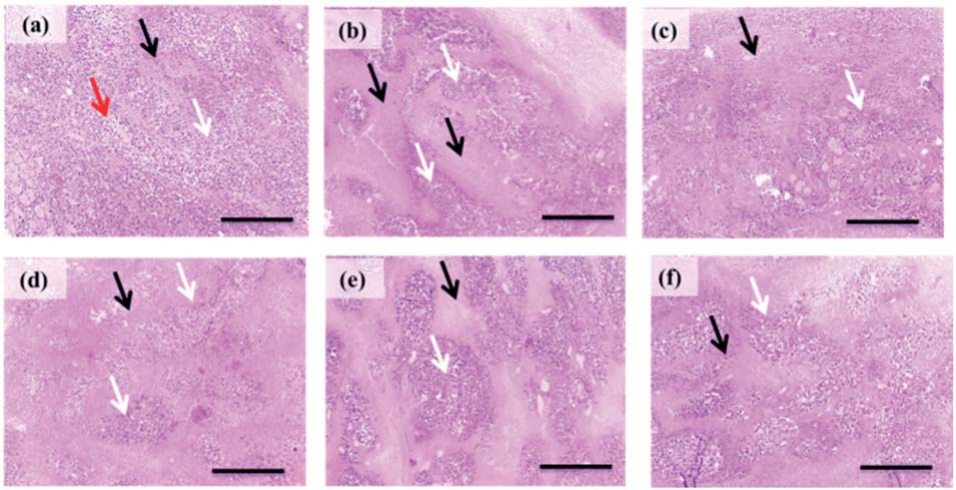
Histopathological pictures of stained EAT tissues with H & E (×100). (a) Section obtained from EAT control group showing numerous large, rounded, and polygonal stained tumor cells (white arrow) aggregated around newly formed blood capillaries (red arrows) innervated by small necrotic areas (black arrow). (b) Section obtained EAT group treated with 5e at 2.5 mg kg^−1^ showing a mild increase of necrotic areas (black arrow). (c) Section obtained from EAT group treated by 5 mg kg^−1^ of 5e showing severe necrosis (black arrow) and an apparent reduction in viable areas (white arrows). (d) Section obtained from EAT mice treated with 5d (2.5 mg kg^−1^) showing a moderate decrease in the size of viable areas (white arrows). (e) Section obtained from EAT mice treated with 5d (5 mg kg^−1^) showing moderate diffuse necrosis (black arrows). (f) Section from doxorubicin-treated group showing a marked decrease of viable areas (white arrows) and an increase of necrotic areas (black arrows).

As shown in (Fig. 6; 5a), light microscope examination of tumor cells of untreated EAT mice revealed wide viable tumor areas interspaced by small necrotic zones along with the appearance of numerous newly formed blood capillaries in the surrounding tissues. EAT mice treated with 5e at 2.5 mg kg^−1^ showed a slight increase in necrotic areas (Fig. 6; 5b) which progressed to severe necrosis and prominent decrease in viable areas at 5 mg kg^−1^ (Fig. 6; 5c). Moderate necrosis was observed in tumor cells of EAT group received 5d at 2.5 mg kg^−1^ (Fig. 6; 5d). Regarding 5d at 5 mg kg^−1^, EAT mice displayed a marked decrease in the number of tumor cells in viable areas accompanied with moderate diffuse necrosis (Fig. 6; 5e).

##### Effect on the survival of EAT mice

**Fig. 7:**
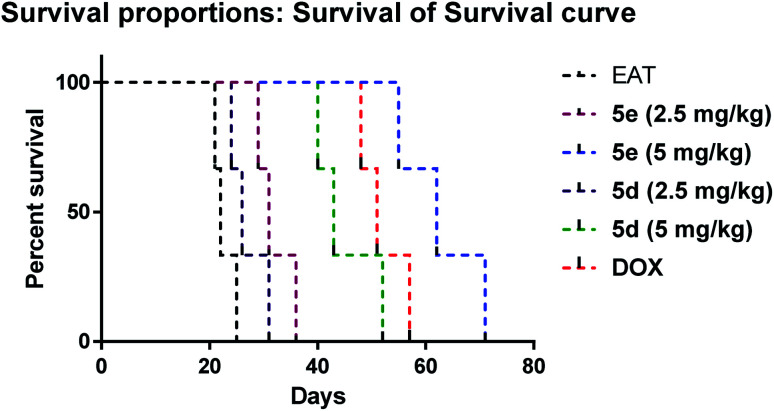
Kaplan–Maeier survival curve of EAT treated groups. Statistical analysis was performed using log-rank (Mantel–Cox) test at *p* < 0.05.

Fig. 7 depicted a significant increase in the lifespan of EAT mice treated with 5e at 2.5 mg kg^−1^ (MST = 31 days), 5e at 5 mg kg^−1^ (MST = 62 days), 5d at 5 mg kg^−1^ (MST = 43 days) and doxorubicin (MST = 51 days) compared to untreated ones (MST = 22 days) at *p* = 0.025. Notably, compound 5e (5 mg kg^−1^) had higher lifespan than doxorubicin treated group, however, the increase of lifespan didn't reach significant difference.

## Experimental

Starting materials were purchased from Sigma-Aldrich (USA), Merck, or Luba and were used with no further purification. Solvents were purchased from Sigma Aldrich or Luba and used without further purification. Reactions were tracked with Thin Layer Chromatography (TLC) using different solvent mixtures as mobile phase and pre-coated aluminum sheets silica gel Merck 60 (F254) as stationary phase; the spots were visualized by exposure to Iodine vapor or UV light under 254 and 366 nm illumination. Melting points were measured on an electrothermal apparatus in closed capillary tubes using Galenkamp melting point apparatus and were uncorrected. Electron impact mass spectra were recorded on direct probe controller inlet part to single quadropole mass analyzer in (Thermo Scientific GCMS) model (ISQ LT) using Thermo X-Calibur software at the regional center for mycology and biotechnology (RCMB) Al-Azhar University, Naser city, Cairo. ^1^H-NMR spectra were recorded in *δ* scale given in ppm and coupling constants *J* are expressed in Hertz on a Bruker 400 MHz spectrophotometer and referred to TMS at center for Drug Discovery and Development Research, Ain Shams University. ^13^CMNR spectra were recorded in *δ* scale given in ppm on a Bruker 400 (at 100 MHz) spectrophotometer at center for Drug Discovery and Development Research, Ain Shams University. Infrared (IR) spectra were measured using KBr discs a FTIR-Prestige 21 Shimadzu infrared spectrophotometer (*ν*_max_ in cm^−1^), with ratio (1 drug: 3KBr) at National Organization of Drug Control and Research (NODCAR). Elemental analysis was performed in Microanalytical center, Al-Azhar University, Egypt. *In vitro* enzyme inhibition assay was implemented in Thermo Fisher Scientific, Life Technologies, United States. *In vitro* cytotoxicity activity was carried out in Nawah Scientific Nawah ScientificInc, Mokatam, Cairo, Egypt. *In vivo* anticancer assessment was established within Faculty of Pharmacy, Mansoura University, Mansoura, Egypt. Adult swiss albino female mice were purchased from the Animal House of Faculty of Pharmacy, Mansoura University (Mansoura, Egypt).

### Chemistry

#### 5-Acetyl-6-methylfuro[2,3-*d*]pyrimidin-4(3*H*)-one (4)

Acetic anhydride (23.8 equiv., 23 ml, 243.3 mmol) was added portion wise to stirred formic acid (29.8 equiv., 46 ml, 1220 mmol) at 0 °C and stirring was continued for 1 h, after which compound (3) (1 equiv., 3.38 g, 20.6 mmol) was added, the ice bath was then removed. The mixture was heated under reflux at 130 °C for 35 hours. The solvent was evaporated by distillation at 160 °C and the crystals obtained were washed with ethanol.

The titled compound was separated golden crystals that turned green upon dryness (74%); m.p. 256 °C; ^1^H-NMR (DMSO-d_6_, 400 MHz): *δ* (ppm) 12.77 (s, 1H, NH D_2_O exchangeable), 8.16 (s, 1H, pyrimidine H), 2.74 (s, 3H, CH_3_), 2.53 (s, 3H, CH_3_ acetyl); FT-IR (*ν*_max_, cm^−1^) 3416 (NH), 3088 (C–H sp^2^), 2926 (C–H sp^3^), 1670 (C=O); MS: (Mwt.: 192): *m*/*z*, 192 [M^+^, (63.39%)] 135.44 (100%); anal. calcd for C_9_H_8_N_2_O_3_: C, 56.25; H, 4.20; N, 14.58; found: C, 56.31; H, 4.34; N, 14.67.

#### General procedure for the synthesis of (*E*)-5-(3-(substituted phenyl)acryloyl)-6-methylfuro[2,3-*d*]pyrimidin-4(3*H*)-one (5a–k)

5-Acetyl-6-methylfuro[2,3-*d*]pyrimidin-4(3*H*)-one (4) (0.192 g, 10 mmol) was dissolved in ethanol (10 ml). To the solution, substituted benzaldehyde (10 mmol) followed by 20% aqueous NaOH solution (1 ml, 5 mmol) was added. The reaction mixture was kept in stirred condition at room temperature for 6–10 h. The reaction mixture was acidified with dilute hydrochloric acid (1 in 10); precipitated solid was filtered and re-crystallized from ethanol. Completion of reaction was monitored on TLC (DCM : ethyl acetate 1 : 1).

#### 5-Cinnamoyl-6-methylfuro[2,3-*d*]pyrimidin-4(3*H*)-one (5a)

The titled compound was separated as yellow crystals (85%); m.p. 224 °C; ^1^H NMR (DMSO-d_6_, 400 MHz): *δ* (ppm) 12.84 (s, 1H, NH D_2_O exchangeable), 8.34 (d, *J* = 15.8 Hz, 1H, βH) 8.19 (s, 1H, pyrimidine H), 7.74 (q, *J* = 6.5 Hz, 2H, ArH), 7.64 (d, *J* = 15.8 Hz, 1H, αH), 7.51–7.42 (m, 3H, ArH), 2.64 (s, 3H, −CH_3_); ^13^C NMR (DMSO-d_6_, 100 MHz): *δ* (ppm); 185.6, 163.9, 158.7, 158.4, 147.6, 142.5, 131.0, 135.4, 129.5 (2C), 129.0 (2C), 128.0, 118.7, 105.4, 14.5; FT-IR (*ν*_max_, cm^−1^) 3418 (NH), 3076 (C–H sp^2^), 2928 (C–H sp^3^), 1670 (C=O), 1595 (C=C); MS: (Mwt.: 280): *m*/*z*, 280 [M^+^, (44.86%)] 41.49 (100%); anal. calcd for C_16_H_12_N_2_O_3_: C, 68.56; H, 4.32; N, 9.99. Found: C, 68.76; H, 4.57; N, 10.23.

#### (*E*)-5-(3-(4-Methoxyphenyl)acryloyl)-6-methylfuro[2,3-*d*]pyrimidin-4(3*H*)-one (5b)

The titled compound was separated as yellow crystals (95%); m.p. 237 °C; ^1^H NMR (DMSO-d_6_, 400 MHz): *δ* (ppm) 12.81 (s, 1H, NH D_2_O exchangeable), 8.31–8.15 (m, 2H, βH, pyrimidine H), 7.7 (d, *J* = 8.7 Hz, 2H, ArH), 7.61 (d, *J* = 15.8 Hz, 1H, αH), 7.02 (d, *J* = 8.7 Hz, 2H, ArH), 3.82 (s, 3H, –OCH_3_), 2.64 (s, 3H, –CH_3_); ^13^C NMR (DMSO-d_6_, 100 MHz): *δ* (ppm); 185.6, 163.9, 161.8, 158.7, 158.4, 147.6, 142.6, 130.8 (2C), 128.0, 125.6, 118.8, 115.0 (2C), 106.5, 55.9, 14.5; FT-IR (*ν*_max_, cm^−1^) 3416 (NH), 3088 (C–H sp^2^), 2931 (C–H sp^3^), 1667 (C=O), 1591 (C=C), 1173 (–OCH_3_); MS: (Mwt.: 310): *m*/*z*, 310 [M^+^, (14.6%)] 119.09 (100%); anal. calcd for C_17_H_14_N_2_O_4_: C, 65.8; H, 4.55; N, 9.09. Found: C, 65.97; H, 4.89; N, 8.92.

#### (*E*)-5-(3-(4-Florophenyl)acryloyl)-6-methylfuro[2,3-*d*]pyrimidin-4(3*H*)-one (5c)

The titled compound was separated as yellow crystals (95%); m.p. 245 °C; ^1^H NMR (DMSO-d_6_, 400 MHz): *δ* (ppm) 12.85 (s, 1H, NH D_2_O exchangeable), 8.32 (d, *J* = 15.8 Hz, 1H, βH) 8.22 (s, 1H, pyrimidine H),7.81 (d, *J* = 8.6 Hz, 2H, ArH), 7.64 (d, *J* = 15.8 Hz, 1H, αH), 7.31 (t, *J* = 8.8 Hz, 2H, ArH), 2.64 (s, 3H, –CH_3_); FT-IR (*ν*_max_, cm^−1^) 3418 (NH), 3086 (C–H sp^2^), 2930 (C–H sp^3^), 1666 (C=O), 1579 (C=C); MS: (Mwt.: 298): *m*/*z*, 298 [M^+^, (58.07%)] 73.15 (100%); anal. calcd for C_16_H_11_FN_2_O_3_: C, 64.43; H, 3.72; N, 9.39. Found: C, 64.23; H, 3.82; N, 9.51.

#### (*E*)-5-(3-(4-Chlorophenyl)acryloyl)-6-methylfuro[2,3-*d*]pyrimidin-4(3*H*)-one (5d)

The titled compound was separated as bright yellow crystals (98%); m.p. 250 °C; ^1^H NMR (DMSO-d_6_, 400 MHz): *δ* (ppm) 12.83 (s, 1H, NH D_2_O exchangeable), 8.38 (d, *J* = 15.8 Hz, 1H, βH) 8.21 (s, 1H, pyrimidine H),7.75 (d, *J* = 8.1 Hz, 2H, ArH), 7.61 (d, *J* = 15.8 Hz, 1H, αH), 7.52 (d, *J* = 8.1 Hz, 2H, ArH), 2.64 (s, 3H, –CH_3_); ^13^C NMR (DMSO-d_6_, 100 MHz): *δ* (ppm) 185.5, 161.9, 158.7, 158.6, 147.6, 141.0, 135.4, 134.4, 130.6 (2C), 129.5 (2C), 128.6, 118.6, 105.40, 14.5; FT-IR (*ν*_max_, cm^−1^) 3396 (NH), 3015 (C–H sp^2^), 2922 (C–H sp^3^), 1686 (C=O), 1605 (C=C); MS: (Mwt.: 314): *m*/*z*, 314/316 [M^+^/M^+^ + 2, (20.09%/2.28%)], 71.06 (100%); anal. calcd for C_16_H_11_ClN_2_O_3_: C, 61.06; H, 3.52; N, 8.90. Found: C, 60.89; H, 3.76; N, 9.13.

#### (*E*)-5-(3-(4-Bromophenyl)acryloyl)-6-methylfuro[2,3-*d*]pyrimidin-4(3*H*)-one (5e)

The titled compound was separated as yellow crystals (97%); m.p. 235 °C; ^1^H NMR (DMSO-d_6_, 400 MHz): *δ* (ppm) 12.87 (s, 1H, NH D_2_O exchangeable), 8.4 (d, *J*= 15.8 Hz, 1H, βH) 8.22 (s, 1H, pyrimidine H),7.7 (d, *J*= 8.8 Hz, 2H, ArH), 7.66(d, *J* = 8.8 Hz, 2H, Ar H), 7.61 (d, *J* = 15.8 Hz, 1H, αH), 2.65 (s, 3H, –CH_3_); ^13^C NMR (DMSO-d_6_, 100 MHz): *δ* (ppm) 185.5, 163.9, 158.8, 158.6, 147.7, 141.1, 134.7, 132.5 (2C), 130.8 (2C), 128.7, 124.2, 118.6, 105.4, 14.45; FT-IR (*ν*_max_ cm^−1^) 3298 (NH), 3030 (C–H sp^2^), 2928 (C–H sp^3^), 1667 (C=O), 1585 (C=C); MS: (Mwt.: 358): *m*/*z*, 358/ 360 [M^+^/M^+^ + 2 (99.84%/ 100%)], 360 (100%); anal. calcd for C_16_H_11_BrN_2_O_3_: C, 53.5; H, 3.09; N, 7.80. Found: C, 53.74; H, 3.38; N, 7.94.

#### (*E*)-5-(3-(4-Nitrophenyl)acryloyl)-6-methylfuro[2,3-*d*]pyrimidin-4(3*H*)-one (5f)

The titled compound was separated as reddish orange crystals (90%); m.p. 287 °C; ^1^H NMR (DMSO-d_6_, 400 MHz): *δ* (ppm) 12.89 (s, 1H, NH D_2_O exchangeable), 8.53 (d, *J* = 15.8 Hz, 1H, βH), 8.29 (d, *J* = 8.6 Hz, 2H, ArH), 8.23 (s, 1H, pyrimidine H),7.98 (d, *J* = 8.7 Hz, 2H, ArH), 7.71 (d, *J* = 15.8 Hz, 1H, αH), 2.66 (s, 3H, –CH_3_); FT-IR (*ν*_max_, cm^−1^) 3298 (NH), 3061 (C–H sp^2^), 2928 (C–H sp^3^), 1665 (C=O), 1591 (C=C); MS: (Mwt.: 325): *m*/*z*, 325 [M^+^, (20.99%)] 72.44 (100%); anal. calcd for C_16_H_11_N_3_O_5_: C, 59.08; H, 3.41; N, 12.92. Found: C, 59.34; H, 3.64; N, 12.84.

#### (*E*)-5-(3-(4-(Dimethylamino)phenyl)acryloyl)-6-methylfuro[2,3-*d*]pyrimidin-4(3*H*)-one (5g)

The titled compound was separated as orange crystals (95%); m.p. above 320 °C; ^1^H NMR (DMSO-d_6_, 400 MHz): *δ* (ppm) 8.8 (d, *J* = 15.6 Hz, 1H, βH) 7.9 (s, 1H, pyrimidine H), 7.58 (d, *J* = 8.7 Hz, 2H, ArH), 7.47 (d, *J* = 15.6 Hz, 1H, αH), 6.73(d, *J* = 8.6 Hz, 2H, Ar H), 2.98 (s, 6H, –N– (CH_3_)_2_), 2.57 (s, 3H, –CH_3_). FT-IR (*ν*_max_, cm^−1^) 3395 (NH), 3048 (C–H sp^2^), 2959 (C–H sp^3^), 1651 (C=O), 1587.4 (C=C); MS: (Mwt.: 323): *m*/*z*, 323 [M^+^, (58.07%)] 65.98 (100%); anal. calcd for C_18_H_17_N_3_O_3_: C, 66.86; H, 5.30; N, 13.00. Found: C, 66.75; H, 5.47; N, 12.88.

#### (*E*)-5-(3-(2,4-Dichlorophenyl)acryloyl)-6-methylfuro[2,3-*d*]pyrimidin-4(3*H*)-one (5h)

The titled compound was separated as yellowish orange crystals (96%); m.p. 269 °C; ^1^H NMR (DMSO-d_6_, 400 MHz): *δ* (ppm) 12.88 (s, 1H, NH D_2_O exchangeable), 8.42 (d, *J* = 15.6 Hz, 1H, βH), 8.22 (s, 1H, pyrimidine H), 7.93 (d, *J* = 8.6 Hz, 1H, ArH), 7.82 (d, *J* = 15.7 Hz, 1H, αH), 7.73 (d, *J* = 2.1 Hz, 1H, ArH), 7.55 (d, *J* = 8.5 Hz, 1H, ArH), 2.66 (s, 3H, –CH_3_). FT-IR (*ν*_max_, cm^−1^) 3424 (NH), 3073 (C–H sp^2^), 2930 (C–H sp^3^), 1655 (C=O), 1589 (C=C); MS: (Mwt.: 348): *m*/*z*, 348 [M^+^, (18.49%)] 310.9 (100%); anal. calcd for C_16_H_10_Cl_2_N_2_O_3_: C, 55.04; H, 2.89; N, 8.02; found: C, 55.23; H, 3.14; N, 8.27.

#### (*E*)-5-(3-(4-Hydroxy-3-methoxyphenyl)acryloyl)-6-methylfuro[2,3-*d*]pyrimidin-4(3*H*)-one (5i)

The titled compound was separated as yellow crystals (31%); m.p. 290 °C; ^1^H NMR (DMSO-d_6_, 400 MHz): *δ* (ppm) 12.88 (s, 1H, NH D_2_O exchangeable), 9.71 (s, 1H, OH D_2_O exchangeable), 8.28–8.15 (m, 2H, βH, pyrimidine H), 7.56 (d, *J* = 15.6 Hz, 1H, αH), 7.36 (s, 1H, ArH), 7.20 (d, *J* = 8.2 Hz, 1H, ArH), 6.84 (d, *J* = 8.1 Hz, 1H, ArH), 3.83 (s, 3H, –OCH_3_), 2.64 (s, 3H, –CH_3_); ^13^C NMR (DMSO-d_6_, 100 MHz): *δ* (ppm) 185.6, 163.8, 158.8, 157.7, 150.1, 148.4, 143.6, 126.9, 124.9, 123.8, 118.8, 116.2, 112.1, 105.6, 56.0, 14.4; MS: (Mwt.: 326): *m*/*z*, 326 [M^+^, (20.09%)] 43.30 (100%); anal. calcd for C_17_H_14_N_2_O_5_: C, 62.57; H, 4.32; N, 8.59. Found: C, 62.48; H, 4.39; N, 8.85.

#### (*E*)-5-(3-(3,4-Dimethoxyphenyl)acryloyl)-6-methylfuro[2,3-*d*]pyrimidin-4(3*H*)-one (5j)

The titled compound was separated as yellow crystals (90%); m.p. 244 °C; ^1^H NMR (DMSO-d_6_, 400 MHz): *δ* (ppm) 8.52 (d, *J* = 15.7 Hz, 1H, βH) 8.12 (s, 1H, pyrimidine H), 7.56 (d, *J* = 15.7 Hz, 1H, αH), 7.4 (s, 1H, ArH), 7.31 (d, *J* = 8.3 Hz, 1H, Ar H), 7.02 (d, *J* = 8.3 Hz, 1H, Ar H), 3.81 (s, 6H, –OCH_3_), 2.63 (s, 3H, –CH_3_); FT-IR (*ν*_max_, cm^−1^) 3306 (NH), 3076 (C–H sp^2^), 2943 (C–H sp^3^), 1678 (C=O), 1591 (C=C); MS: (Mwt.: 340): *m*/*z*, 340 [M^+^, (49.21%)] 200 (100%); anal. calcd for C_18_H_16_N_2_O_5_: C, 63.52; H, 4.74; N, 8.23; found: C, 63.42; H, 4.81; N, 8.37.

#### (*E*)-6-Methyl-5-(3-(3,4,5-trimethoxyphenyl)acryloyl)furo[2,3-*d*]pyrimidin-4(3*H*)-one (5k)

The titled compound was separated as yellowish orange crystals (85% yield); m.p. 140 °C; ^1^H NMR (DMSO-d_6_, 400 MHz): 12.93 (s, 1H, NH D_2_O exchangeable), 8.36 (d, *J* = 15.6 Hz, 1H, βH) 8.16 (s, 1H, pyrimidine H), 7.55 (d, *J* = 15.6 Hz, 1H, αH), 7.10 (s, 2H, ArH), 3.82(s, 6H, O-CH_3_), 3.71 (s, 3H, O-CH_3_) 2.63 (s, 3H, –CH_3_); ^13^C NMR (DMSO-d_6_, 100 MHz): *δ* (ppm) 185.7, 163.9, 158.8, 158.2, 153.6, 147.5, 143.1, 140.1, 131.0, 127.4, 118.7, 106.5, 105.5, 60.6, 56.4, 14.5; FT-IR (*ν*_max_, cm^−1^) 3250 (NH), 3059 (C–H sp^2^), 2945 (C–H sp^3^), 1684 (C=O), 1580 (C=C); MS: (Mwt.: 370): *m*/*z*, 370 [M+, (22.16%)] 139.14 (100%); anal. calcd for C_17_H_14_N_2_O_5_: C, 62.57; H, 4.32; N, 8.59. Found: C, 62.48; H, 4.39; N, 8.85.

### Biological evaluation

#### *In vitro* anti-proliferative activity against 60 cell line panel

The NCI *in vitro* anticancer screening is a two-stage process, beginning with all compounds assessment against the full NCI 60 cell lines panel representing leukemia, NSCLC, melanoma, colon cancer, CNS cancer, breast cancer, ovarian cancer, renal cancer and prostate cancer at a single dose of 10 μM. The output from the single dose screen is reported as a mean graph.

#### *In vitro* cell cycle analysis by flow cytometry

Cell cycle analysis by flow cytometry was carried out for the two chalcone based compounds (5d, 5e) due to their broad anticancer spectrum, at Nawah Scientific Inc, (Mokatam, Cairo, Egypt), on MCF7 breast cancer cell line, which is derived from mammary gland, breast; metastatic site: pleural effusion, human (ATCC® HTB-22 ™) served as the cells' source in ATCC-formulated Dulbecco's Modified Eagle's Medium, (ATCC # 30-2003).

#### *In vitro* cytotoxicity activity assay

Doxorubicin, a known anti-cancer agent, was used as positive control compound. The MCF-7-ADR doxorubicin resistant breast cancer cell line was obtained from Nawah Scientific Inc., (Mokatam, Cairo, Egypt). Cells were maintained in DMEM media supplemented with 100 mg ml^−1^ of streptomycin, 100 units per ml of penicillin and 10% of heat-inactivated fetal bovine serum in humidified, 5% (v/v) CO_2_ atmosphere at 37 °C.

#### *In vivo* anticancer activity assessment

Adult Swiss albino female mice weighing 15–20 g were used in this study. They were purchased from the Animal House of Faculty of Pharmacy, Badr University in Cairo (Cairo, Egypt). Mice were kept in polypropylene cages in a controlled temperature and humidity condition (23 ± 1 °C, 40–60% humidity), with 12 h light–dark alternating cycles and allowed free access to standard mice pellet diet and water ad libitum. Mice were then left for initial adaptation period of one week before the experiment. The experimental protocols were permitted by the Research Ethics Committee of the Faculty of Pharmacy, Badr University in Cairo (Approval no. PC-105-A) and performed in agreement with the recommendations published by the US National Institutes of Health for the proper care and use of laboratory animals (NIH Publication No. 85-23, revised 1996).

#### Acute toxicity study of 5e and 5d (LD_50_ determination)

According to the method described by Lorke (1983),^24^ LD_50_ was determined through two steps including a total of 13 adult male Swiss albino mice (weighing 150–200 gm). Mice were fasted for 18 h before the experiment. The first step involved 9 mice (for each drug) divided into 3 groups (3 mice each) which received 3 single increasing doses of 5e and 5d (10, 100, 1000 mg kg^−1^; i.p, respectively). Mice were then observed for 24 h to estimate any change of behavior and mortality. Based on the first step results, the second step involved different 4 mice (for each compound) allocated into 4 groups (one mouse each) which received 4 subsequent doses of 5e and 5d (Table 3). Animals were then observed for 24 h to monitor their behavior as well as mortality. Depending on the results of the second step, LD50 was determined as follows;
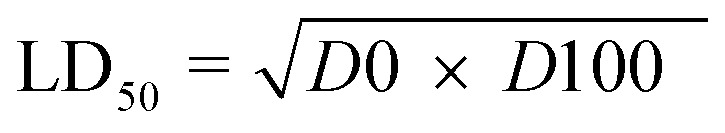
where, *D*0 = highest dose that didn't cause mortality. *D*100 = lowest dose that caused mortality.

#### Induction of Ehrlich ascites tumor (EAT)

Ehrlich ascites carcinoma (EAC) swiss albino mouse was obtained from National Cancer Institute (NCI), Cairo University (Cairo, Egypt). Ascitic tumor cells were maintained in mice by intraperitoneal inoculation to another mouse every 10 day. EAT fluids were drawn from tumor-bearing mice at the log phase (7–8 days of tumor growth), suspended, and diluted with PBS. About 0.2 ml of tumor suspension containing 2 × 10^6^ tumor cells were afterwards transplanted subcutaneously into tested groups.^25^ Five days later, EAT was developed as confirmed by formation of palpable mass (50–100 mm^3^).

#### Experimental design

Mice were set into 6 groups (8 mouse each) as follows; untreated Ehrlich ascites tumor control group (EAT) and treated EAT groups that received either compound 5e (5 & 2.5 mg kg^−1^; i.p. daily), compound 5d (5 & 2.5 mg kg^−1^; i.p. daily), or doxorubicin (DOX; 2 mg kg^−1^; i.p. daily) (LC laboratories, Cat. No. 25316-40-9). Treatment compounds were dissolved in 5% DMSO and injected intraperitoneally at the 5^th^ day (first day of treatment = day 0) after subcutaneous implantation of EAT for 20 days. EAT mice control group received DMSO (1 ml kg^−1^; i.p/daily).

#### Tumor volume and tumor growth inhibition (TGI) estimation

The change in tumor volume was monitored every 5 days from the first day of treatment (day 0) till day 20. The tumor diameter of each mouse was measured using a digital vernier caliper and according to the following formula, tumor volume (TV) was calculated.

TV = length (mm) × [width (mm)]2 × 0.52, tumor growth inhibition (TGI) was calculated as follows; TGI (%) = 1 − (RTV of the treated group at the day of measurement)/(RTV of control group at the day of measurement) × 100. RTV = (tumor volume at the day of measurement)/tumor volume at the initial day).

After 3 weeks of treatment, mice were sacrificed by cervical dislocation and tumor masses were isolated, washed with ice cold saline, dried and weighed. For each mouse, tumor index was calculated by dividing tumor weight by total body weight measured at the end of experiment (day 20). Three mice from each group were maintained for determining median survival time (MST) and increase in lifespan. Isolated tumor tissues were then fixed in 10% formalin for histopathological examination and immunohistochemistry investigation.

#### Immunohistochemistry analysis of caspase-3

EAT sections embedded in paraffin were dewaxed, hydrated with xylene, and immersed in EDTA antigen retrieval (pH 8). Specimens were then blocked with tris-buffered saline (TBS) and 5% bovine serum albumin (BSA) at room temperature for 1 h, followed by incubation with primary antibody specific to caspase-3 over night at 4 °C. Slides were afterwards washed with TBS and incubated with goat anti-rabbit IgG secondary antibody (Servicebio, China) for 30 min at room temperature. Sections were then washed with TBS and visualized using diaminobenzidine (DAB) (Liquid DAB + Substrate Chromogen System; Dako, Denmark). Mayer's hematoxylin (Sigma-Aldrich, Inc. USA) was used finally for counterstaining. Percentage (%) of caspase-3 immunopositive areas (distinct brown color) to total area was determined through selecting 5 non-overlapping fields (magnification ×100) in each section (mean ± S.E.M of 5 fields). Stained tumor sections images were analyzed using full HD microscopic imaging system linked to Leica application suite (Leica Microsystems GmbH, Germany).

#### Oxidative stress biomarkers

About 0.2 gm of tumor tissue from each mouse was used for colorimetric determination of oxidative stress biomarkers namely, total antioxidant capacity (TAC), glutathione (GSH), and malondialdehyde (MDA) using commercially available kits (Biodiagnostic ®).

#### Histopathological examination

On day 21, animals were sacrificed and tumor specimens were excised, weighed and fixed in Slices of tumor tissues were immersed and fixed in 10% neutral-buffered formalin. Graded series of ethanol were used for dehydration of tumor specimens which were afterwards cleared in xylene and embedded in paraffin wax. Sections of 4-μm thickness from paraffin-embedded samples were then used for staining by hematoxylin & eosin (H & E) and examined under light microscope.

#### Statistical analysis

Data were expressed as mean ± S.E.M. and statistically assessed by GraphPad Prism (GraphPad, San Diego, CA) using one-way analysis of variance (ANOVA), followed by Tukey's multiple comparison test. Difference in tumor volume measured at different days for each group was performed using Two-way ANOVA, followed by Tukey's multiple comparison test. Kaplan–Meier method and log-rank (Mantel–Cox) test were used for constructing and measuring the significance of survival between groups. Significance between groups was considered at *p* < 0.05.

## Conclusion

Novel furo[2,3-*d*]pyrimidine based chalcone derivatives were synthesized and evaluated for theirs *in vitro* anti-proliferative activity against NCI 60 cell line panel. The halogen bearing compounds 5d and 5e presented potent anti-proliferative activity and were selected for further *in vitro* and *in vivo* biological activity. *In vitro* five doses NCI 60 cell panel assay revealed potent GI_50_s values by both 5d and 5e, against the MCF-7 breast cancer cell line; 1.39 μM 0.505 μM, respectively. Therefore, compounds 5d and 5e were tested for their cytotoxic activity against the resistant MCF-7 cell line and demonstrated lower IC_50_s (1.2 μM ± 0.21, 1.9 μM ± 0.32, respectively) when compared to doxorubicin (3.3 μM ± 0.18). Consequently, *in vivo* anticancer activity was performed, enlightening 5e comparable anticancer activity against EAT to doxorubicin. This was demonstrated through testing compound 5e at a dose of 5 mg kg^−1^ for its influence on the tumor growth inhibition ratio, immunoexpression of caspase-3, oxidative stress biomarkers and EAT survival.

## Conflicts of interest

There are no conflicts to declare.

## Supplementary Material

RA-012-D2RA00889K-s001
