# Supplementary material for: Identification of novel furo[2,3-d]pyrimidine based chalcones as potent anti-breast cancer agents: synthesis, in vitro and in vivo biological evaluation
Source: RSC Adv. 2022 Mar 15;12(13):8193–201. doi: 10.1039/d2ra00889k (PMC8982411; doi:10.1039/d2ra00889k)
Supplement: RA-012-D2RA00889K-s001 [file RA-012-D2RA00889K-s001.pdf]

Electronic Supplementary Material (ESI) for RSC Advances.
This journal is © The Royal Society of Chemistry 2022

Identification of novel furo[2,3-*d*]pyrimidine based chalcones as potent anti-breast cancer agents: synthesis, *in vitro* and *in vivo* biological evaluation

Results and Discussion

Table 1: NCI 60 cancer cell lines cell growth percentage exhibited by some of the investigated final compounds (5a-k)

Panel/ Cell line	Cell Growth Percent for the tested compounds										
	**5a**	**5b**	**5c**	**5d**	**5e**	**5f**	**5g**	**5h**	**5i**	**5j**	**5k**
Leukemia											
CCRF-CEM	NA	NA	NA	NA	6.65	NA	104.43	NA	NA	56.73	34.04
HL-60(TB)	99.24	79.95	57.06	-47.76	-4.98	63.95	82.21	7.83	116.51	73.98	65.94
K-562	97.71	88.17	45.39	-63.22	10.79	57.7	86.84	17.79	110.71	64.14	45.17
MOLT-4	103.34	101.79	101.25	40.71	8.42	95.28	92.86	86.84	103.84	84.46	68.35
RPMI-8226	118.62	104.52	73.78	-14.13	-3.59	72.24	97.8	28.4	121.96	77.23	61.89
SR	113.77	65.79	57.18	-30.62	NA	41.43	NA	32.97	125.99	NA	NA
Non-Small Cell Lung Cancer											
A549/ATCC	101.52	91.91	86.67	31.72	9.19	96.02	92.71	78.72	100.97	85.18	81.65
EKVX	110.94	98.11	76.06	61.97	19.74	97.93	88.69	117.24	97.94	73.76	61.91
HOP-62	93.41	109.97	96.6	6.15	12.07	96.56	105.98	92.59	93.95	106.51	101.35
HOP-92	95.7	98.57	65.42	-4.23	-10.66	78.81	86.44	95.54	116.27	88.39	96.38
NCI-H226	91.17	101.99	92.57	93.99	-8.79	94.9	92.37	107.44	101.59	90.56	92.12
NCI-H23	95.21	86.11	80.21	39.75	-11.17	89.38	91.11	89.98	98.73	77.79	68.91
NCI-H322M	98.81	92.1	89.29	45.25	2.39	89.1	97	92.37	98.28	83.01	85.84
NCI-H460	101.9	95.6	89.62	21.01	3.92	97.83	104.09	93.4	103.46	94.94	83.68
NCI-H522	97.84	84.69	69.65	17.51	13.9	81.1	93.66	39.06	100.66	70.94	47.66
Colon Cancer											
COLO 205	106.57	108.07	107.24	70.83	3.83	100.25	104.38	107.08	102	106.32	95.35
HCC-2998	102.82	100.98	102.27	83.09	-33.93	97.86	103.56	102.05	100.16	110.01	112.17

HCT-116	100.99	79.34	59.2	13.73	-11.63	82.67	99.98	36.35	102.26	71.21	61.28
HCT-15	102.95	80.09	66.72	19.92	-27.43	73.3	95.08	50.56	98.07	60.48	49.32
HT29	117.69	97.92	82.81	12.91	15.27	92.15	99.07	30.39	107.96	98.6	90.68
KM12	106.19	76.18	71.61	13.59	-5.18	78.54	99.76	52.07	103.07	98.54	96.94
SW-620	107.21	111.57	97.09	8.49	3.84	84.79	113.98	31.2	100.02	96.16	77.52
CNS Cancer											
SF-268	102.68	89.07	89.14	50.11	5.39	93.94	97.26	94.61	99.57	82.2	81.29
SF-295	105.39	102.88	101.59	77.03	14.15	102.91	96.07	104	99.26	104.9	103.99
SF-539	100.98	97.94	95.75	14.53	-9.63	91.11	94.78	93.33	97.53	96.48	95.74
SNB-19	96.94	96.28	85	42.01	23.47	87.15	95.56	83.98	97.26	90.78	93.63
SNB-75	71.45	84.33	39.85	-11.42	-2.44	69.9	100.92	44.61	84.37	69.52	77.97
U251	104.27	96.29	85.84	24.28	12.05	88.4	90.54	85.08	103.44	NA	NA
Melanoma											
LOX IMVI	91.96	87.72	68.87	8.44	-90.64	70.43	98.25	52.24	97.73	80.09	66.16
MALME-3M	97.71	95.59	80.74	71.03	24.45	91.63	95.31	99.64	103.06	82.35	88.07
M14	97.52	95.42	93.77	82.29	15.37	83.51	99.94	93.89	99.58	76.8	75.33
MDA-MB-435	99.76	88.6	72.4	6.74	19.77	88.45	100.44	57.17	102.31	77.9	74.4
SK-MEL-2	108.01	92.56	87.27	55.75	11.95	101.38	95.11	91.28	109.13	83.55	82.53
SK-MEL-28	106.33	100.04	91.71	39.38	23.88	95.88	95.83	89.07	101.05	94.98	93.9
SK-MEL-5	96.47	88.92	71.45	37.48	8.55	93.61	96.43	81.65	101.44	89.3	89.88
UACC-257	NA	NA	NA	NA	32.71	NA	95.02	NA	96.57	71.89	73.05
UACC-62	94.43	83.8	71.29	18.18	13.91	80.95	89.01	53.5	96.5	77.66	86.97
Ovarian Cancer											
IGROV1	86.12	96.84	71	38.93	8.79	82.79	96.95	89.22	100.42	81.08	75.38
OVCAR-3	106.18	97.66	75.24	4.32	-10.86	82.06	101.83	48.57	107.76	98.1	77.82
OVCAR-4	98.18	88.87	79.06	29.78	35.99	88.75	114.31	68.6	101.05	86.57	74.95
OVCAR-5	109.42	119.58	124.69	34.53	22.34	103.4	94.86	133.71	96.48	100.39	96.5
OVCAR-8	97.57	91.14	71.1	15.79	7.33	87.14	98.99	45.43	103.38	87.33	79.48
NCI/ADR-RES	99.23	94.87	69.73	10.43	-30.5	79.13	104.12	42.98	100.64	89.65	82.13
SK-OV-3	100.5	NA	95.45	NA	31.47	NA	105.55	NA	102.67	102.11	98.66
Renal Cancer											

786-0	102.7	100.01	97.12	29.69	11.46	90.85	98.23	83.77	101.52	98.31	94.25
A498	106.22	105.35	94.26	62.41	23.74	90.75	94.74	94.3	94.79	82.93	93.43
ACHN	98.33	90.52	66.95	27.18	1.43	82.24	103.7	66.41	101.26	87.53	74.68
CAKI-1	86.24	75.72	49.46	26.06	28.45	50.18	96.49	46.13	87.15	84.27	72.7
RXF 393	NA	NA	NA	NA	4.34	NA	100.38	NA		88.54	66.26
SN12C	99.51	92.43	82.62	25.41	1.76	86.6	101.17	67.03	97.24	87.53	79.84
TK-10	178.11	143.95	132.38	32.29	40.89	119.78	98.41	123.43	135	160.57	150.85
UO-31	75.33	88.53	61.89	37.95	19.36	64.2	87.3	67.58	84.29	67.29	63.17
Prostate Cancer											
PC-3	100.78	96.64	76.25	34.23	25.46	90.62	101.04	59.15	102.54	86.77	83.44
DU-145	104.96	86.71	66.58	6.76	5.67	80.29	103.23	78.22	115.94	81.19	64.83
Breast Cancer											
MCF7	83.98	69.42	33.9	15.37	9.43	61.04	83.93	38.96	90.21	60.51	47.89
MDA-MB-231/ATCC	90.91	97.31	80.04	22.35	16.1	95.53	102.11	89.02	106.43	92.23	86.67
HS 578T	96.44	101.93	96.2	60.55	24.3	106.62	102.67	101.89	93.01	93.49	84.32
BT-549	100.6	99.36	88.71	20.23	2.55	95.3	92	73.75	100.21	84.7	72.6
T-47D	NA	NA	NA	NA	4.69	NA	102.73	NA	NA	71.98	58.91
MDA-MB-468	102.11	91.64	18.16	10.59	-55.38	81.37	91.23	86.73	113.44	70.98	64.34
MEAN % Growth	101.09	94.21	79.16	**26.93**	**5.49**	85.81	97.43	73.07	102.26	86.06	79.38

Table 2: GI50 values of compounds 5d and 5e against 59 NCI cell line

**Panel/ Cell line**	**GI50 (µM)**	
**Cell line**	**Compound 5d**	**Compound 5e**
Leukemia		
CCRF-CEM	1.81	1.01
HL-60(TB)	2.63	2.32
K-562	1.82	0.798
MOLT-4	2.75	2.84
RPMI-8226	1.66	1.36

SR	1.51	1.35
Non-Small Cell Lung Cancer		
A549/ATCC	2.66	2.73
EKVX	2.37	2.37
HOP-62	2.83	2.62
HOP-92	1.47	1.35
NCI-H226	1.96	2.48
NCI-H23	2.51	2.07
NCI-H322M	2.32	2.39
NCI-H460	2.56	3.25
NCI-H522	1.79	1.89
Colon Cancer		
COLO 205	1.9	1.7
HCC-2998	1.79	1.7
HCT-116	1.59	1.26
HCT-15	1.29	1.09
HT29	2.9	2.36
KM12	2.97	1.51
SW-620	2.53	2.16
CNS Cancer		
SF-268	3.17	3.08
SF-295	4.5	4.22
SF-539	2.37	1.73
SNB-19	3.28	1.8
SNB-75	------	1.23
U251	1.97	1.44
Melanoma		
LOX IMVI	1.6	1.03
MALME-3M	2.13	1.89
M14	1.96	2.14
MDA-MB-435	2.4	2.00
SK-MEL-2	3.13	2.34
SK-MEL-28	2.51	2.39
SK-MEL-5	2.25	1.56
UACC-257	2.72	2.73
UACC-62	1.64	1.33
Ovarian Cancer		
IGROV1	1.82	1.81
OVCAR-3	2.3	1.85

OVCAR-4	4.43	2.51
OVCAR-5	3.17	2.93
OVCAR-8	2.67	2.08
NCI/ADR-RES	2.44	1.84
SK-OV-3	3.09	4.08
Renal Cancer		
786-0	3.02	1.8
A498	1.48	2.46
ACHN	1.99	1.6
CAKI-1	3.06	1.34
RXF 393	1.97	1.41
SN12C	2.79	2.95
TK-10	5.09	4.46
UO-31	1.09	0.781
Prostate Cancer		
PC-3	2.49	2.36
DU-145	2.82	2.03
Breast Cancer		
MCF7	1.39	0.505
MDA-MB-231/ATCC	2.65	3.47
HS 578T	3.66	3.49
BT-549	2.24	1.74
T-47D	1.72	1.86
MDA-MB-468	1.38	-----
**Mean GI50**	**2.41**	**1.23**

Five doses Curves

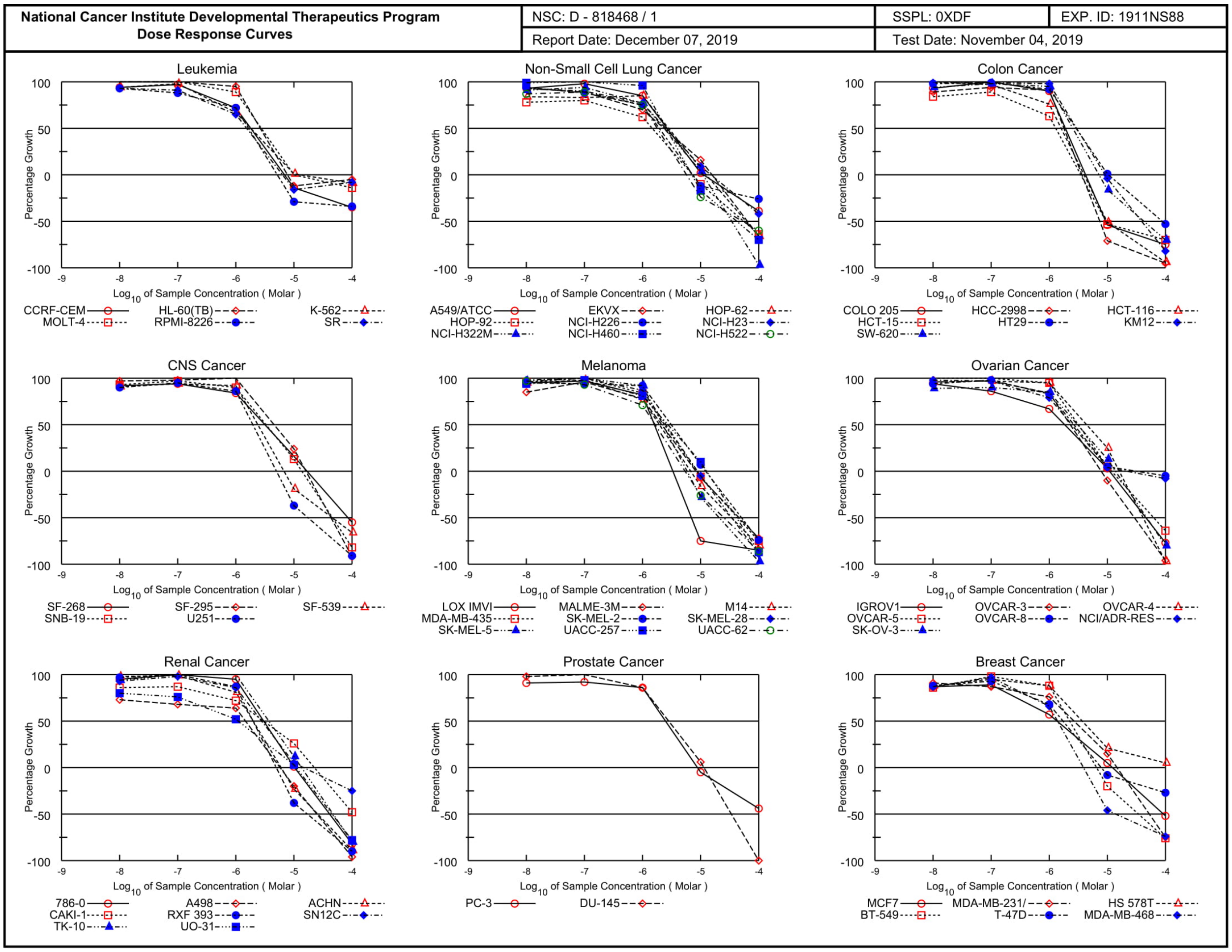

Figure 1: Dose response curve for compound 5d in 9 Cell panels (Leukemia, Melanoma, NSLC, Colon, CNS, Ovarian, renal, prostate and breast cancers)

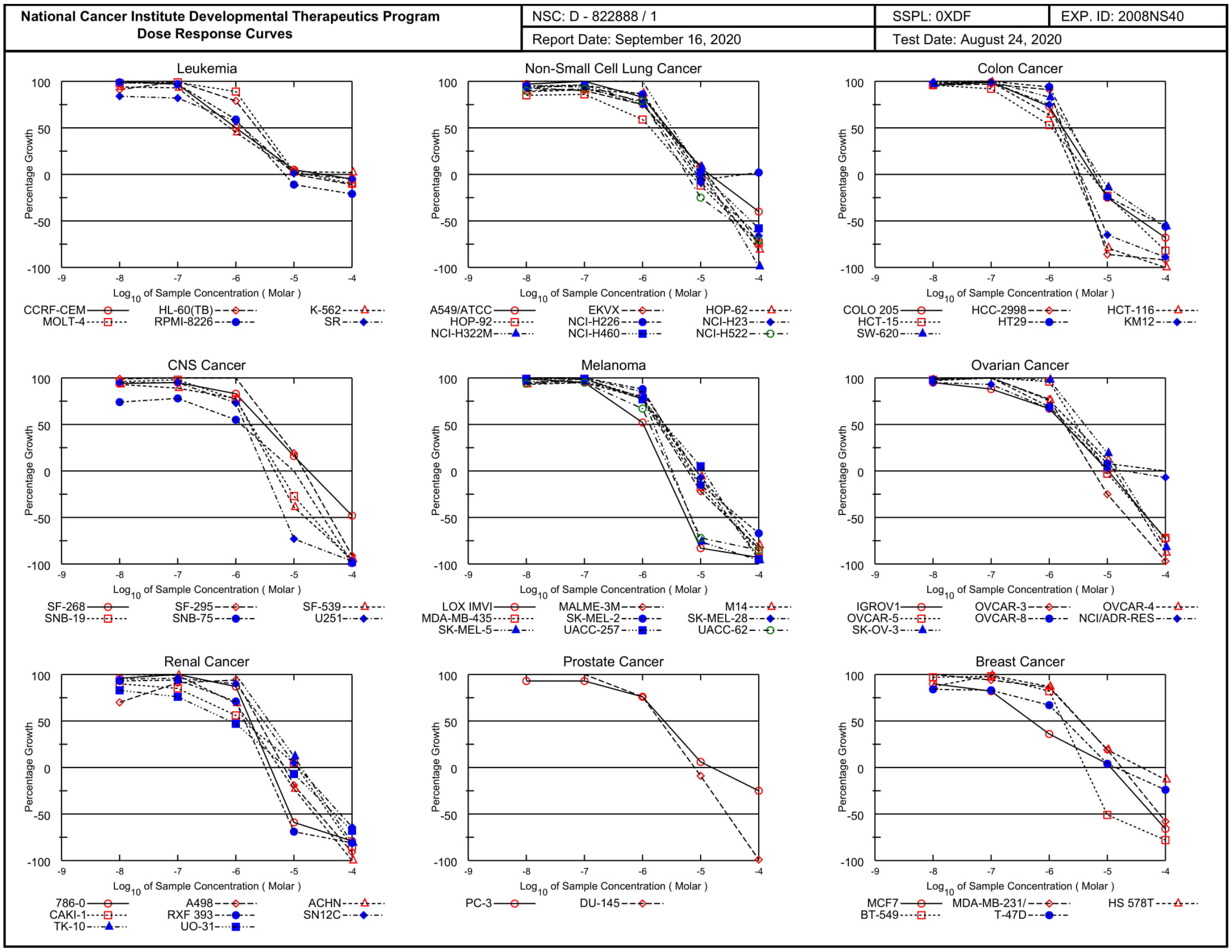

Figure 2: Dose response curve for compound 5e in 9 Cell panels (Leukemia, Melanoma, NSLC, Colon, CNS, Ovarian, renal, prostate and breast cancers)

Experimental

Chemistry and analysis

Supplementary Data NMR charts

. 5-Acetyl-6-methylfuro[2,3-*d*]pyrimidin-4(3H)-one (4)

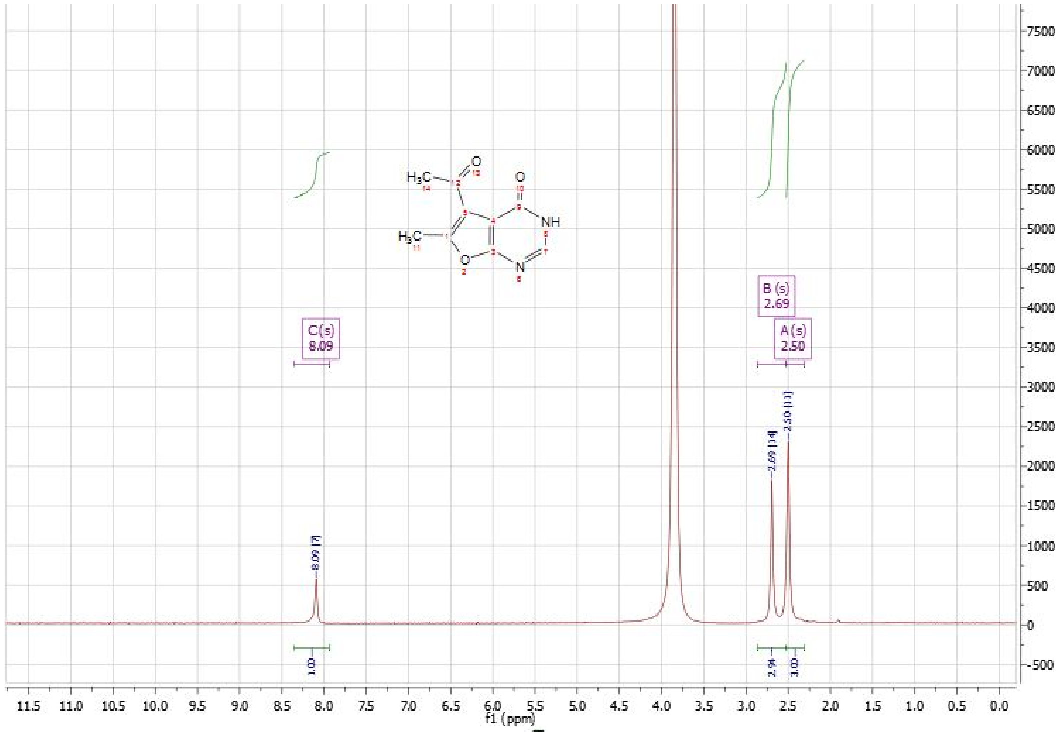

5-Cinnamoyl-6-methylfuro[2,3-*d*]pyrimidin-4(3H)-one (5a)

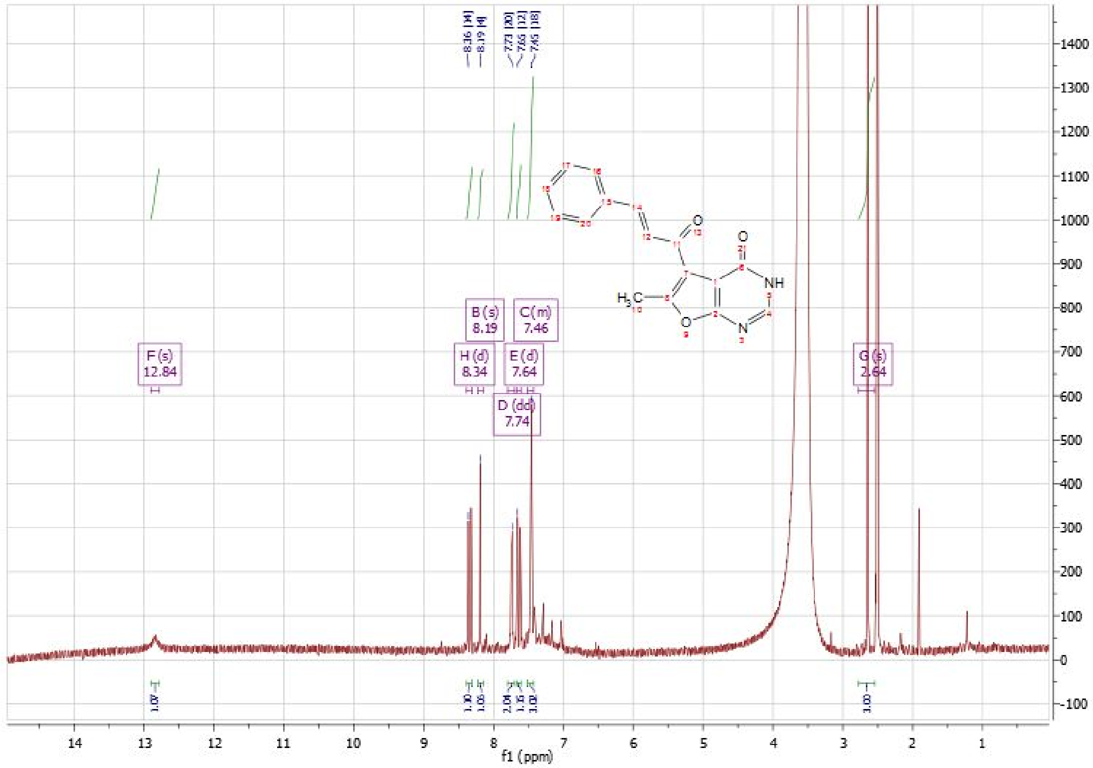

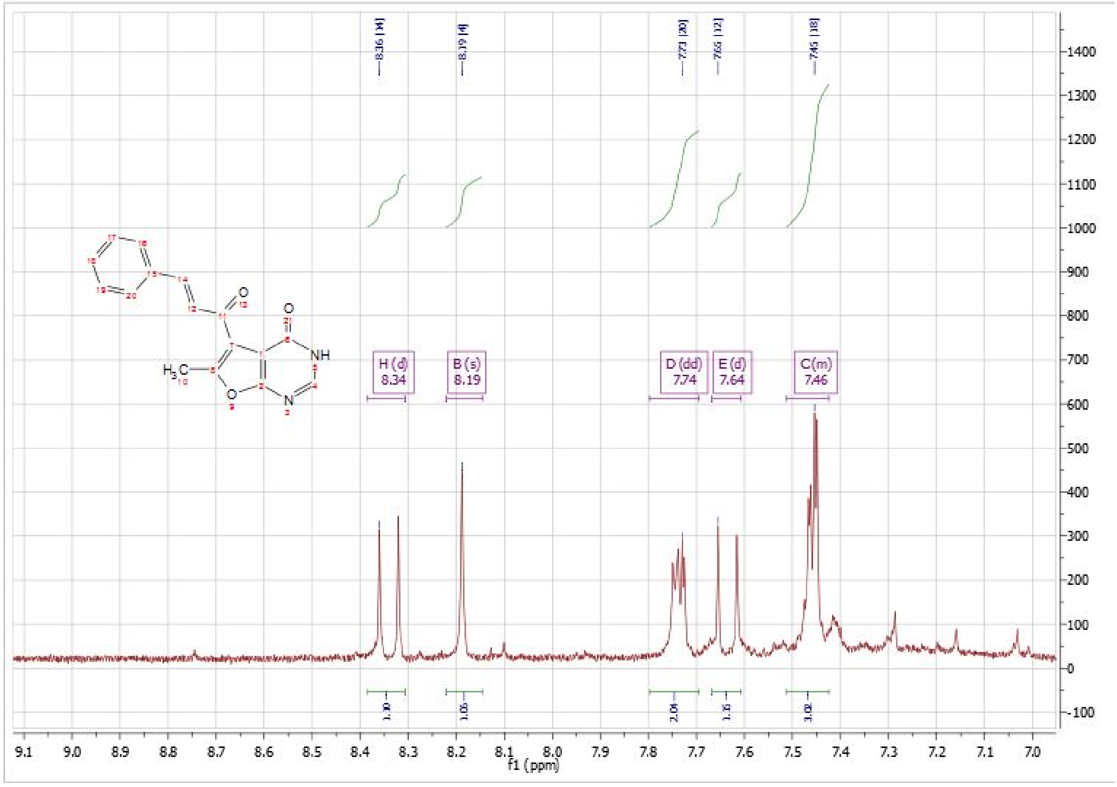

8.36
8.19 [4]
7.73 [20]
7.65 [12]
7.45 [18]
H (d) 8.34
B (s) 8.19
D (dd) 7.74
E (d) 7.64
C (m) 7.46
1.00
1.06
2.04
1.16
3.02
NH
O
H_3C
N
f1 (ppm)

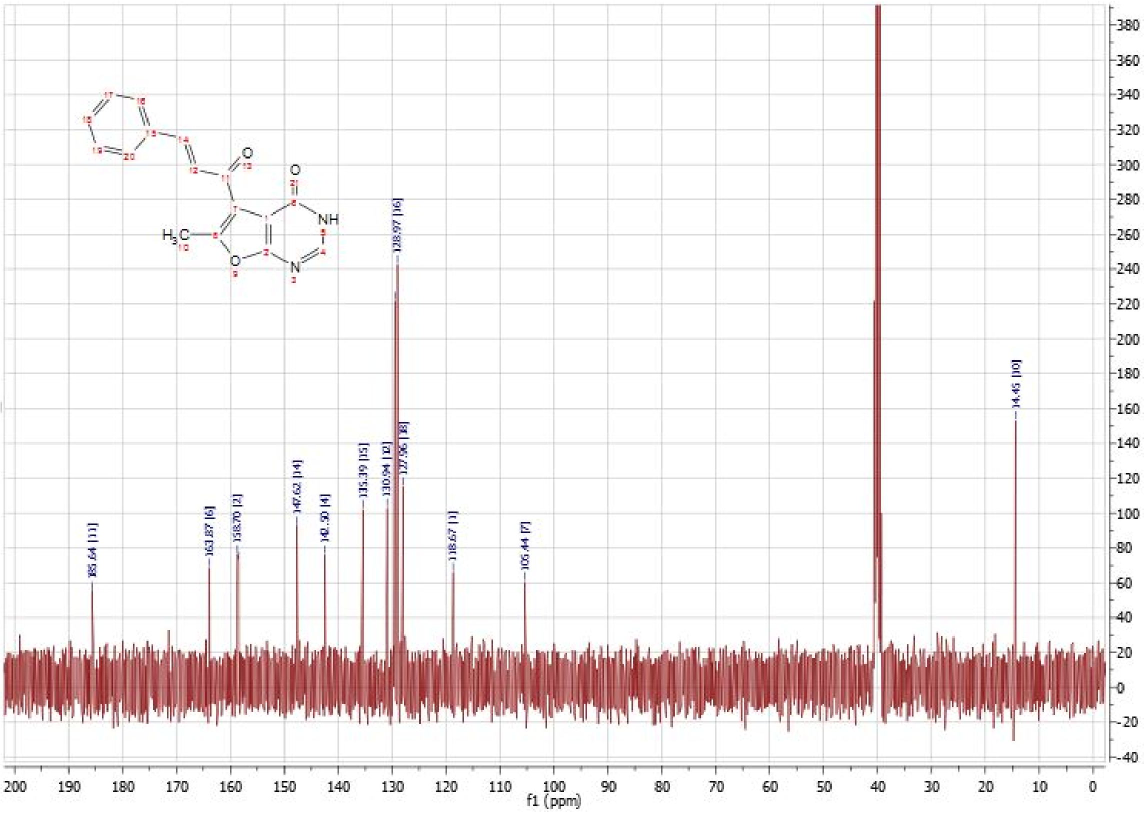

185.64 [11]
163.87 [6]
158.70 [2]
147.62 [14]
135.39 [15]
130.94 [12]
128.97 [16]
127.96 [18]
118.67 [1]
105.44 [7]
14.45 [10]
NH
O
H_3C
N
f1 (ppm)

(E)-5-(3-(4-methoxyphenyl)acryloyl)-6-methylfuro[2,3-*d*]pyrimidin-4(3H)-one (5b)

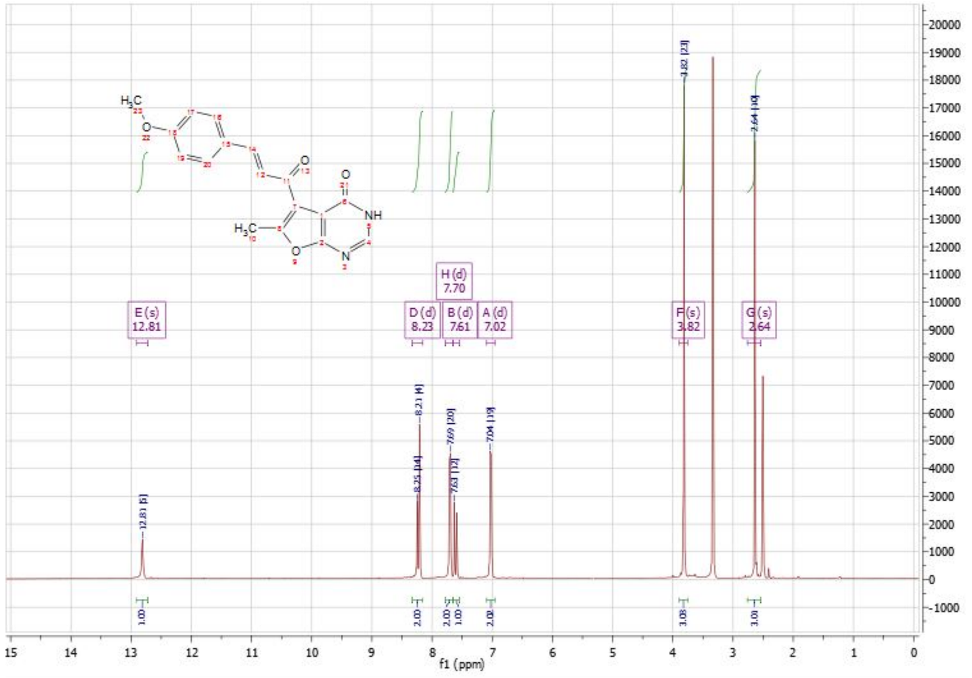

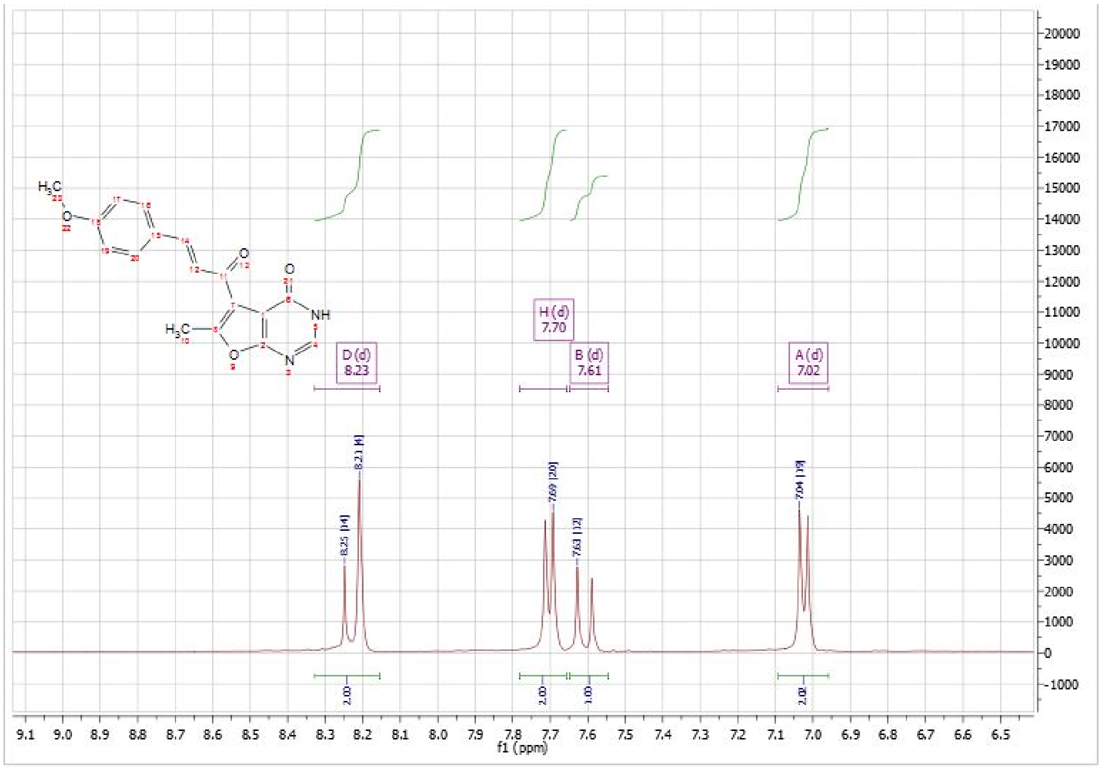

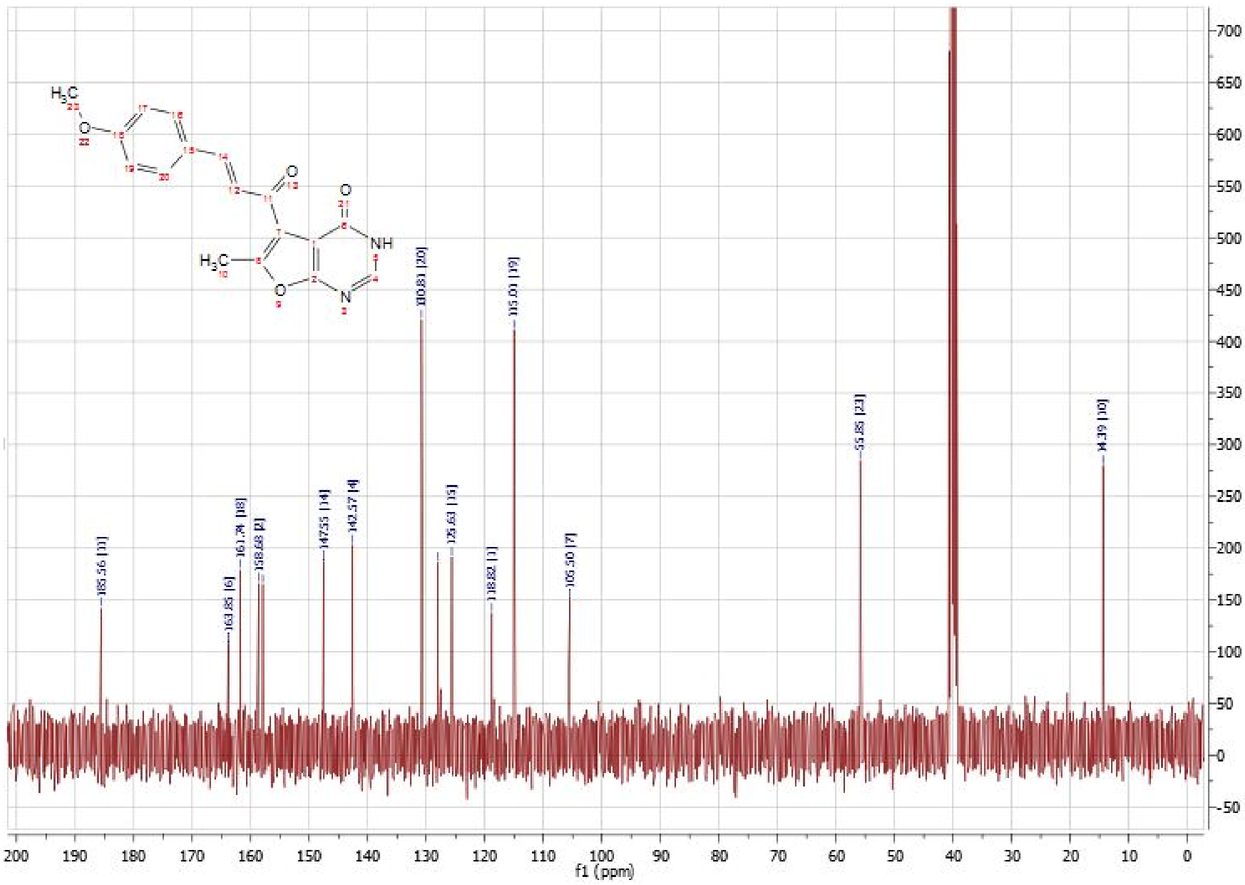

(E)-5-(3-(4-florophenyl)acryloyl)-6-methylfuro[2,3-*d*]pyrimidin-4(3H)-one (5c)

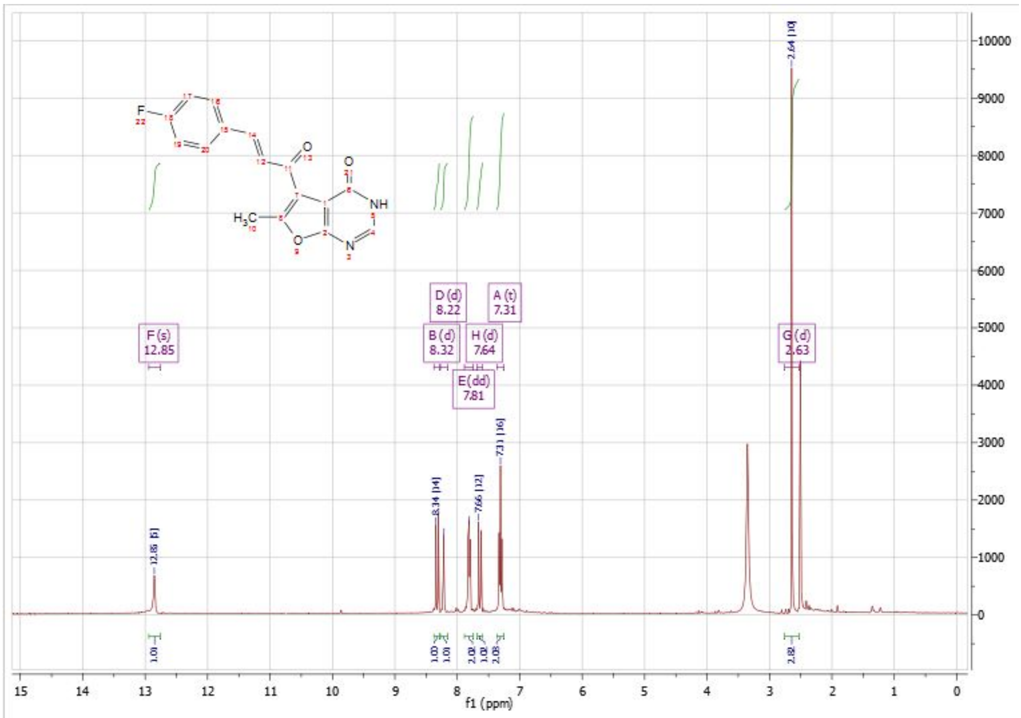

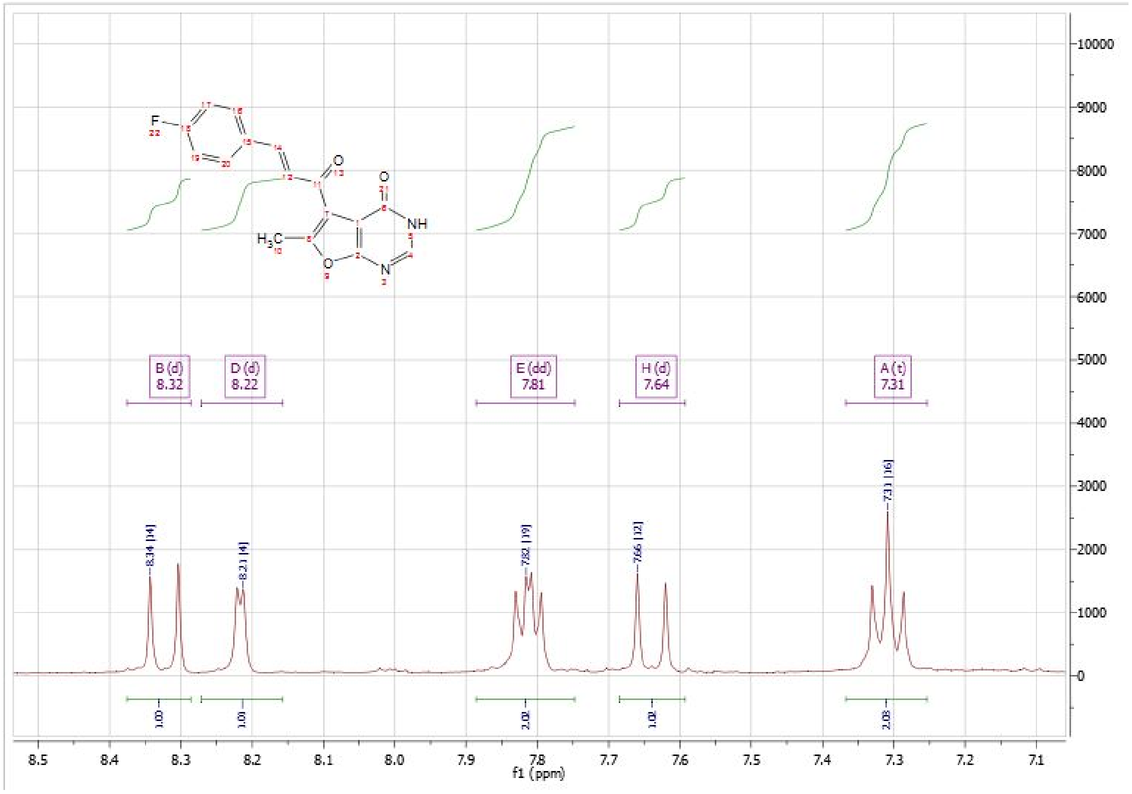

(E)-5-(3-(4-chlorophenyl)acryloyl)-6-methylfuro[2,3-*d*]pyrimidin-4(3H)-one (5d)

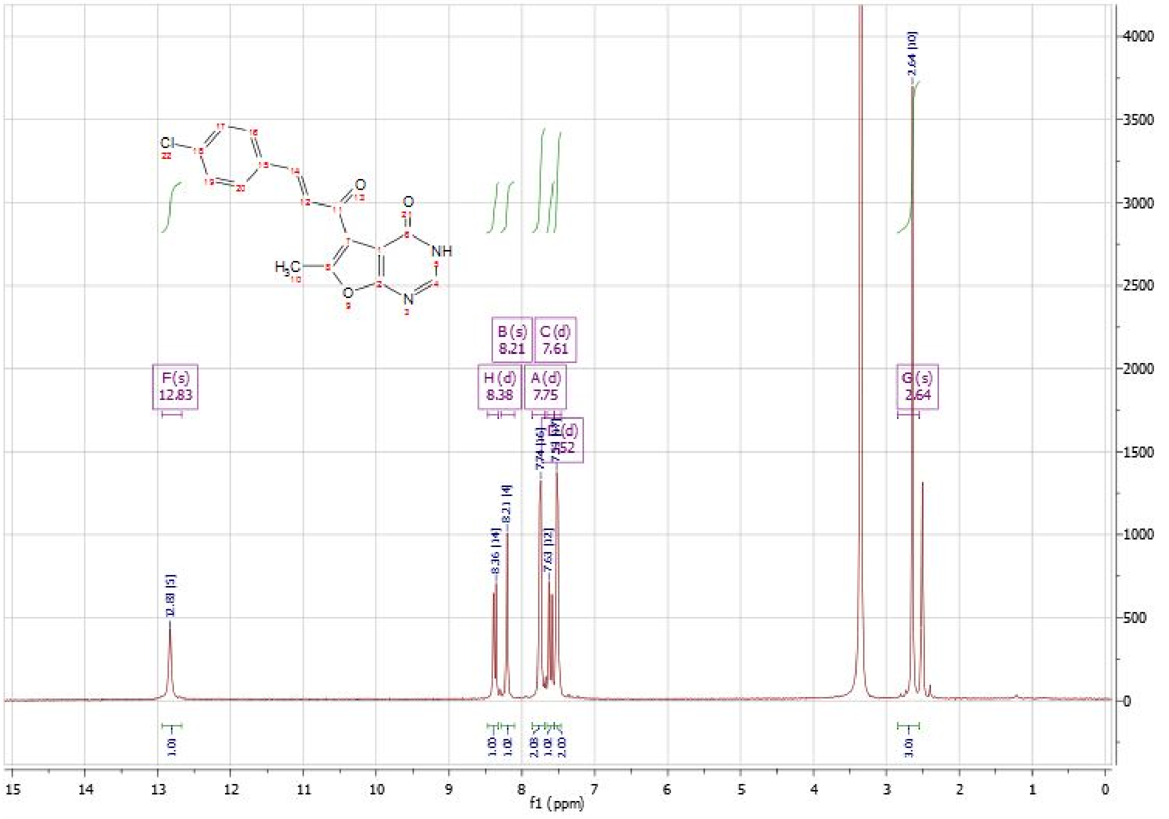

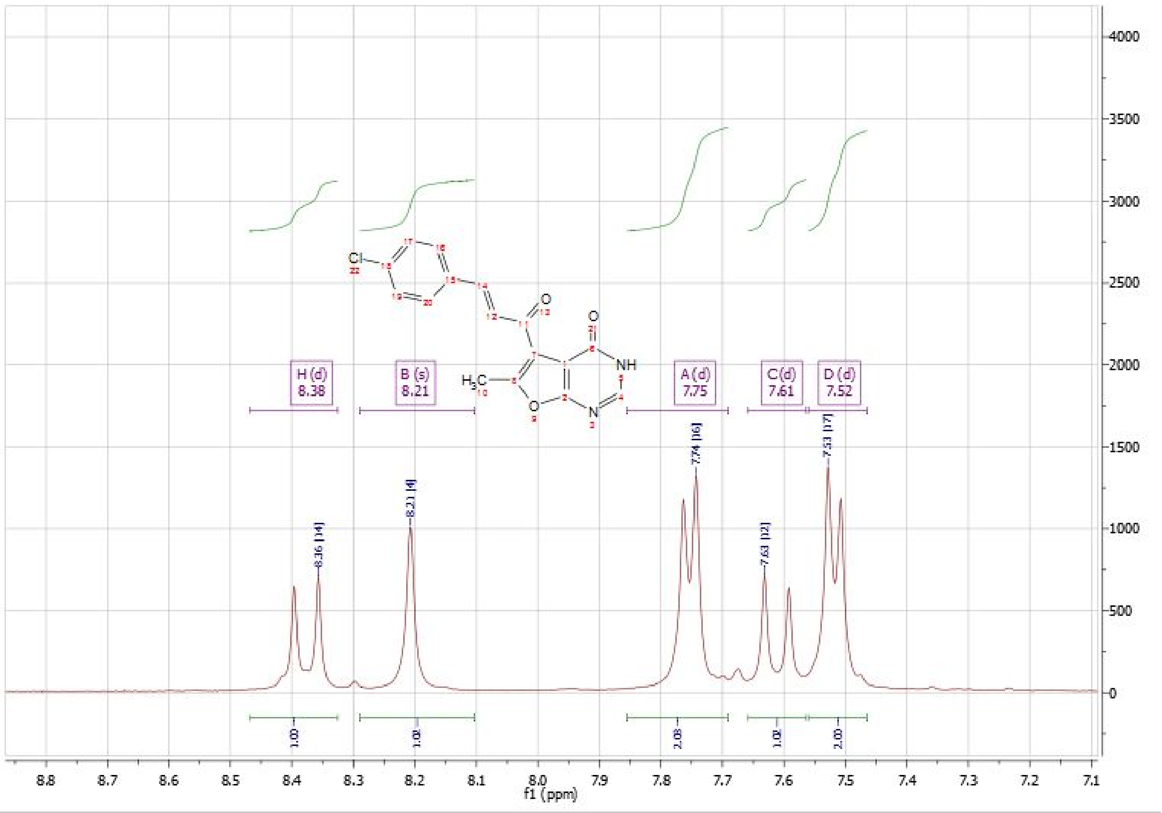

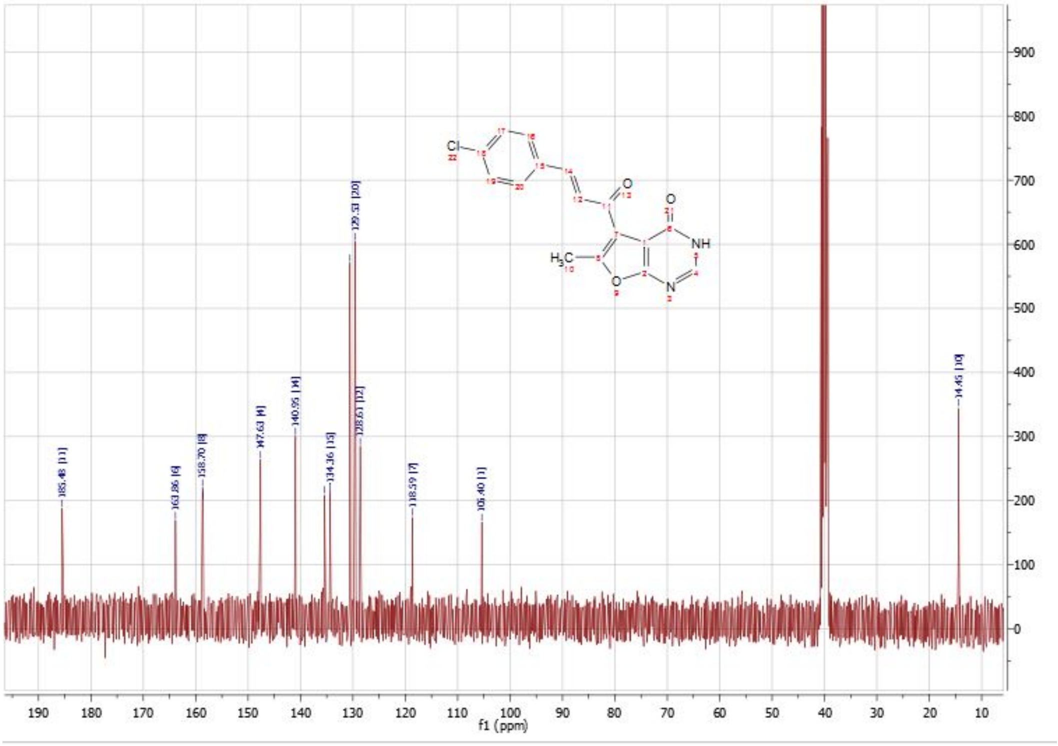

(E)-5-(3-(4-Bromophenyl)acryloyl)-6-methylfuro[2,3-*d*]pyrimidin-4(3H)-one. (5e)

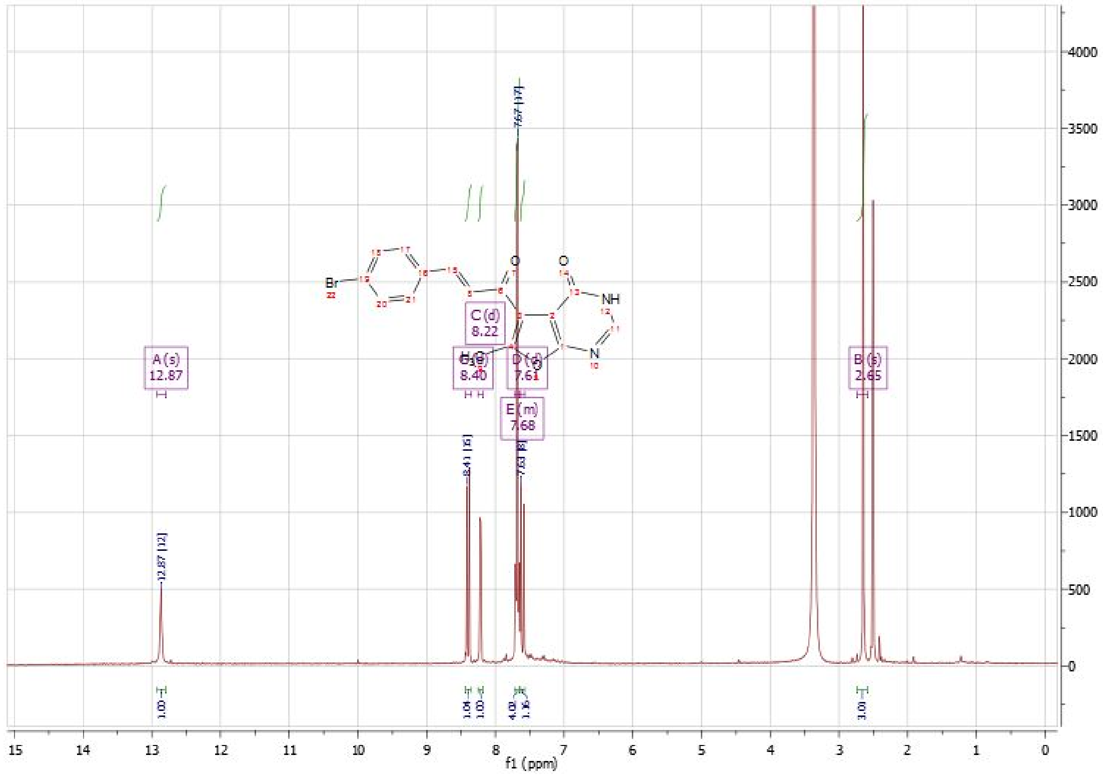
A (s)
12.87
B (s)
2.65
C (d)
8.22
D (d)
7.61
E (m)
7.68
f1 (ppm)

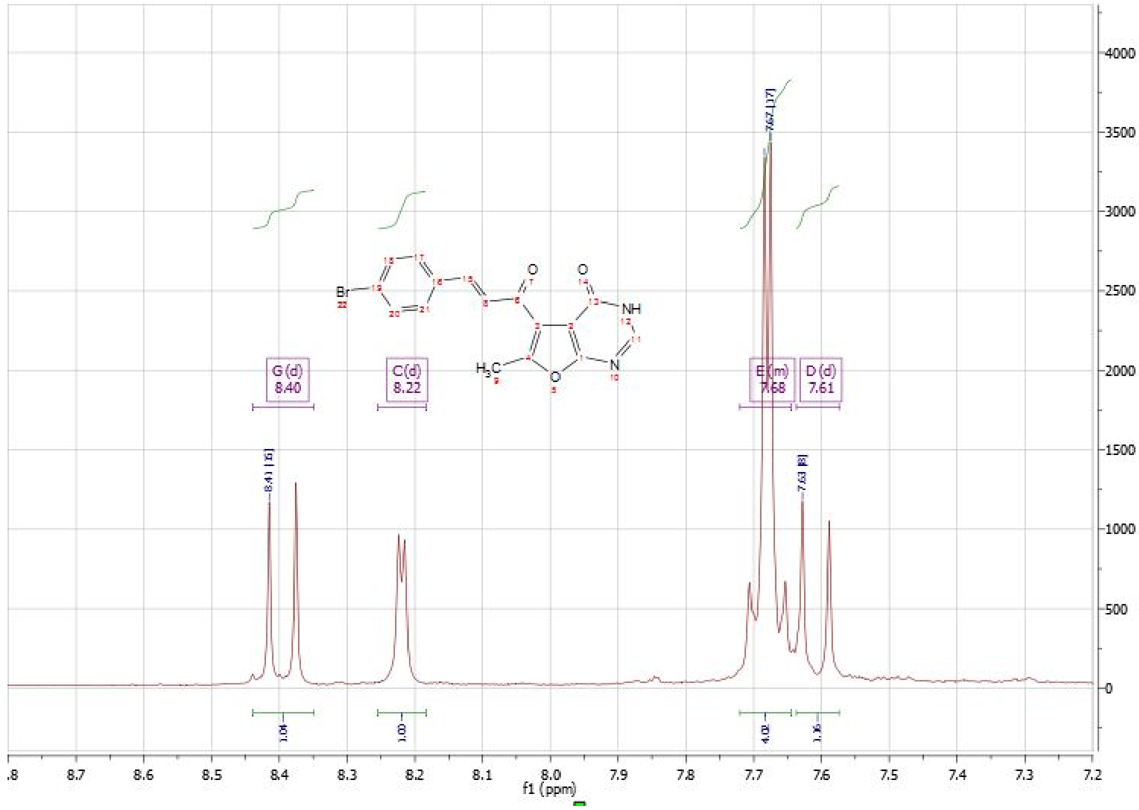
G (d)
8.40
C (d)
8.22
E (m)
7.68
D (d)
7.61
f1 (ppm)

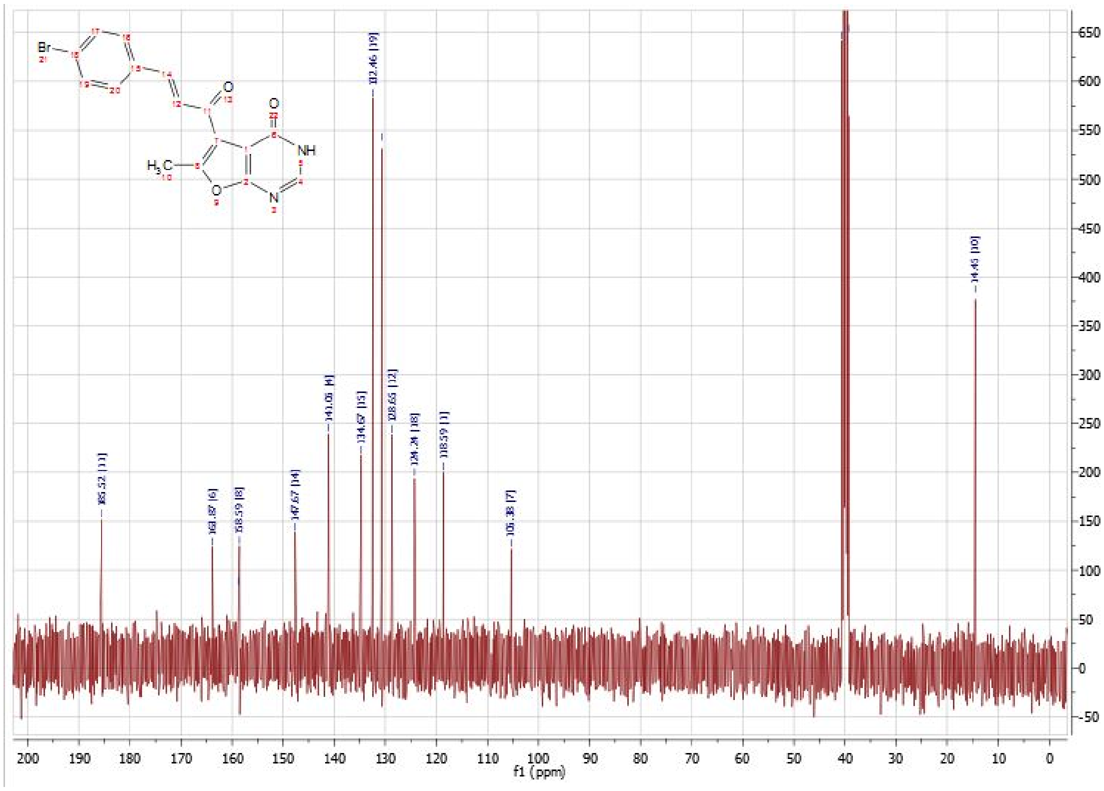

(E)-5-(3-(4-Nitrophenyl)acryloyl)-6-methylfuro[2,3-*d*]pyrimidin-4(3H)-one (5f)

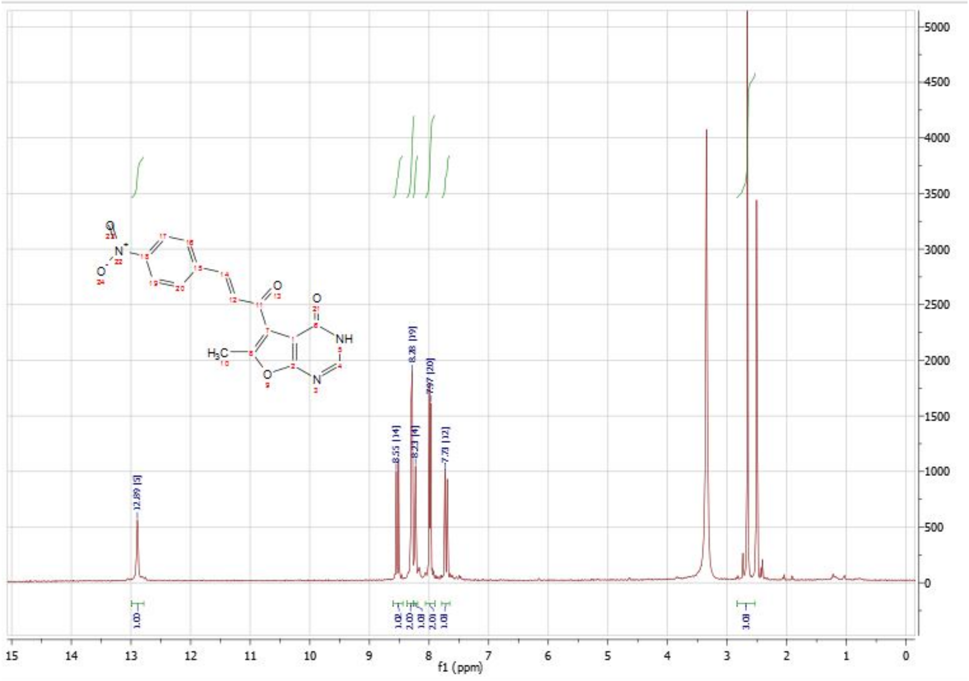

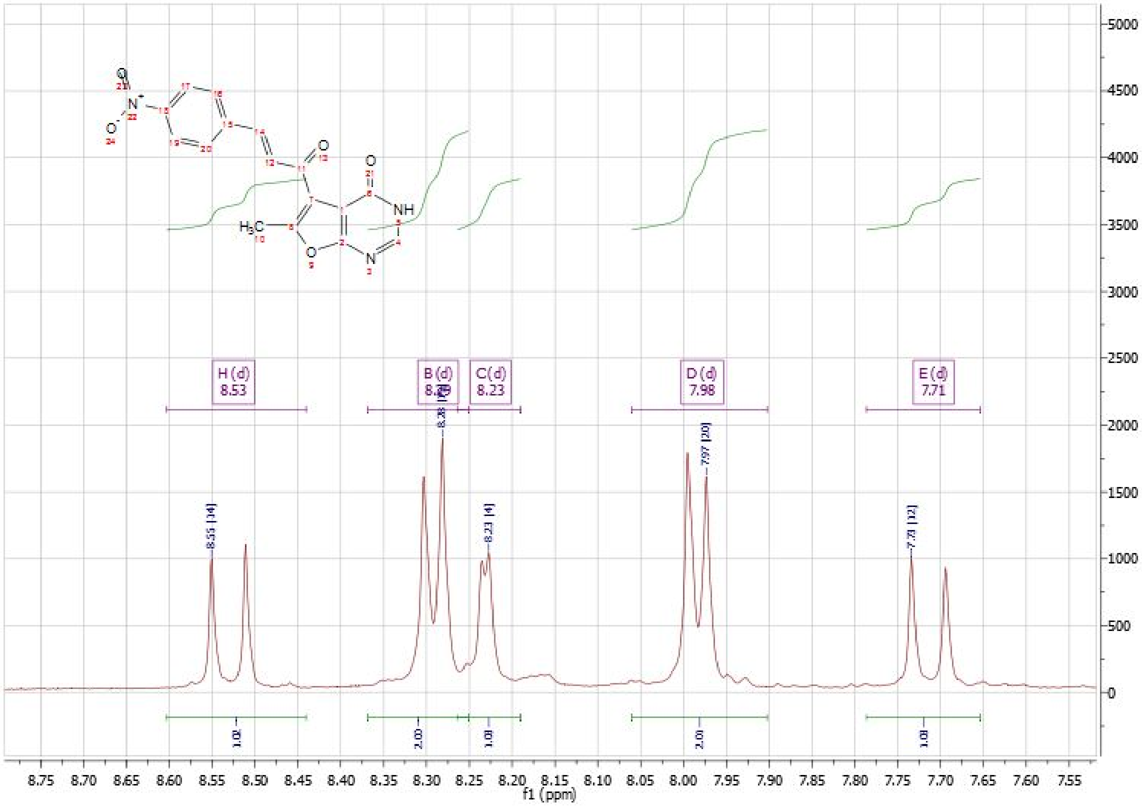

(E)-5-(3-(4-(dimethylamino)phenyl)acryloyl)-6-methylfuro[2,3-*d*]pyrimidin-4(3H)-one (5g)

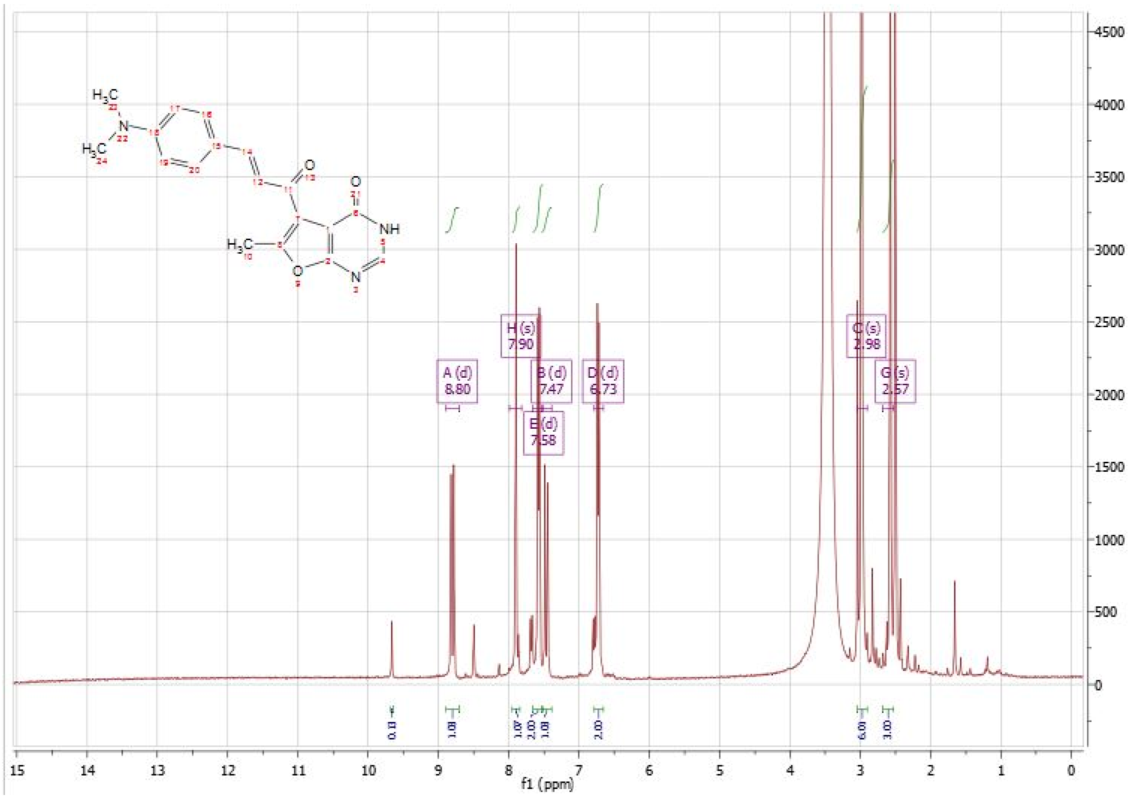

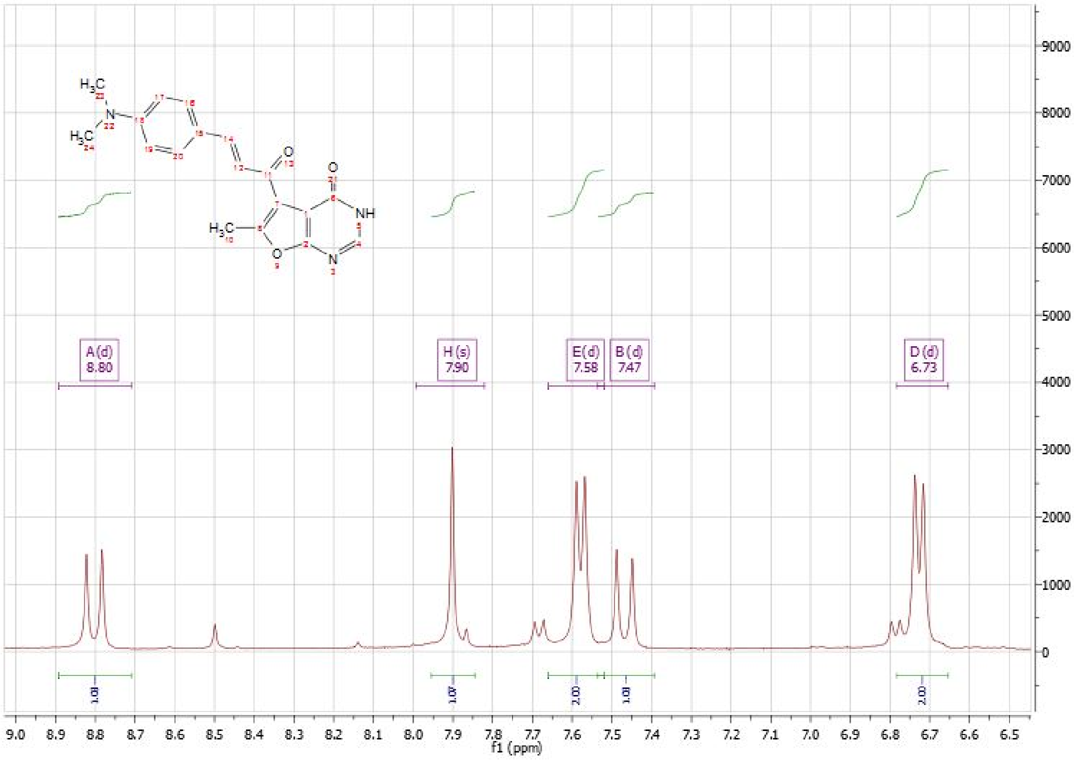

(E)-5-(3-(2,4-dichlorophenyl)acryloyl)-6-methylfuro[2,3-*d*]pyrimidin-4(3H)-one (5h)

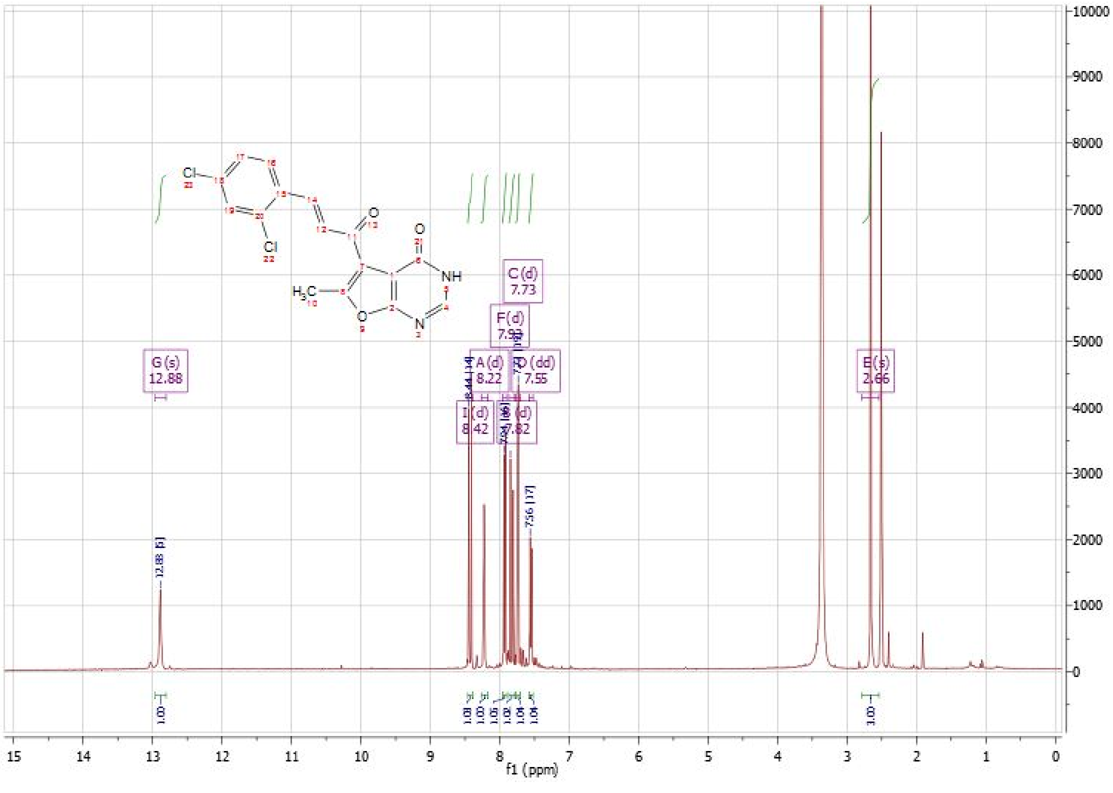

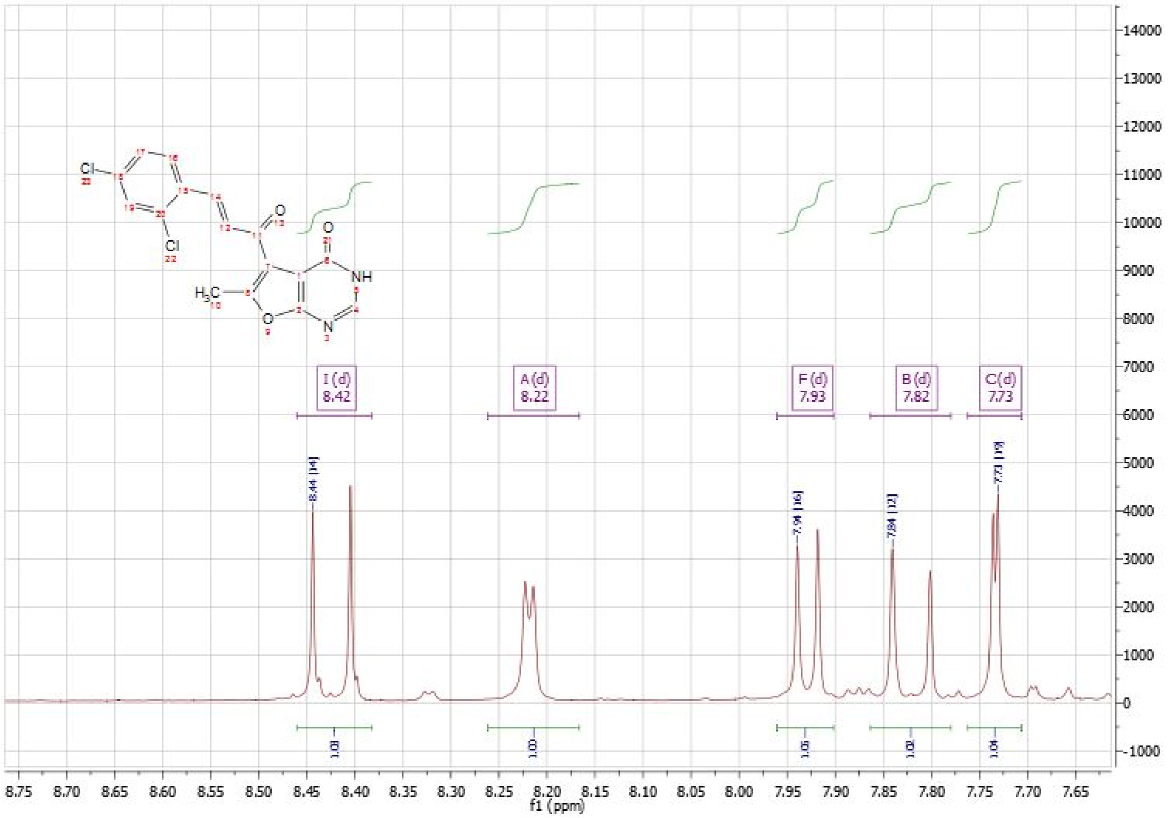

(E)-5-(3-(4-hydroxy-3-methoxyphenyl)acryloyl)-6-methylfuro[2,3-*d*]pyrimidin-4(3H)-one (5i)

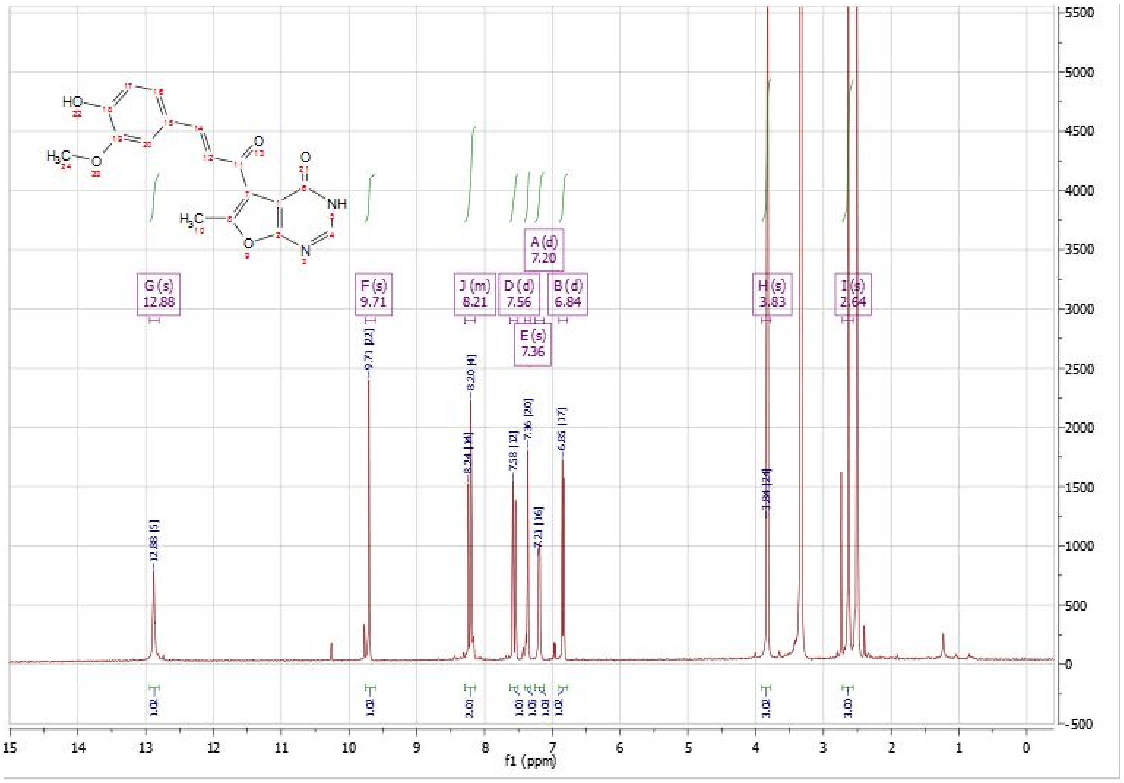

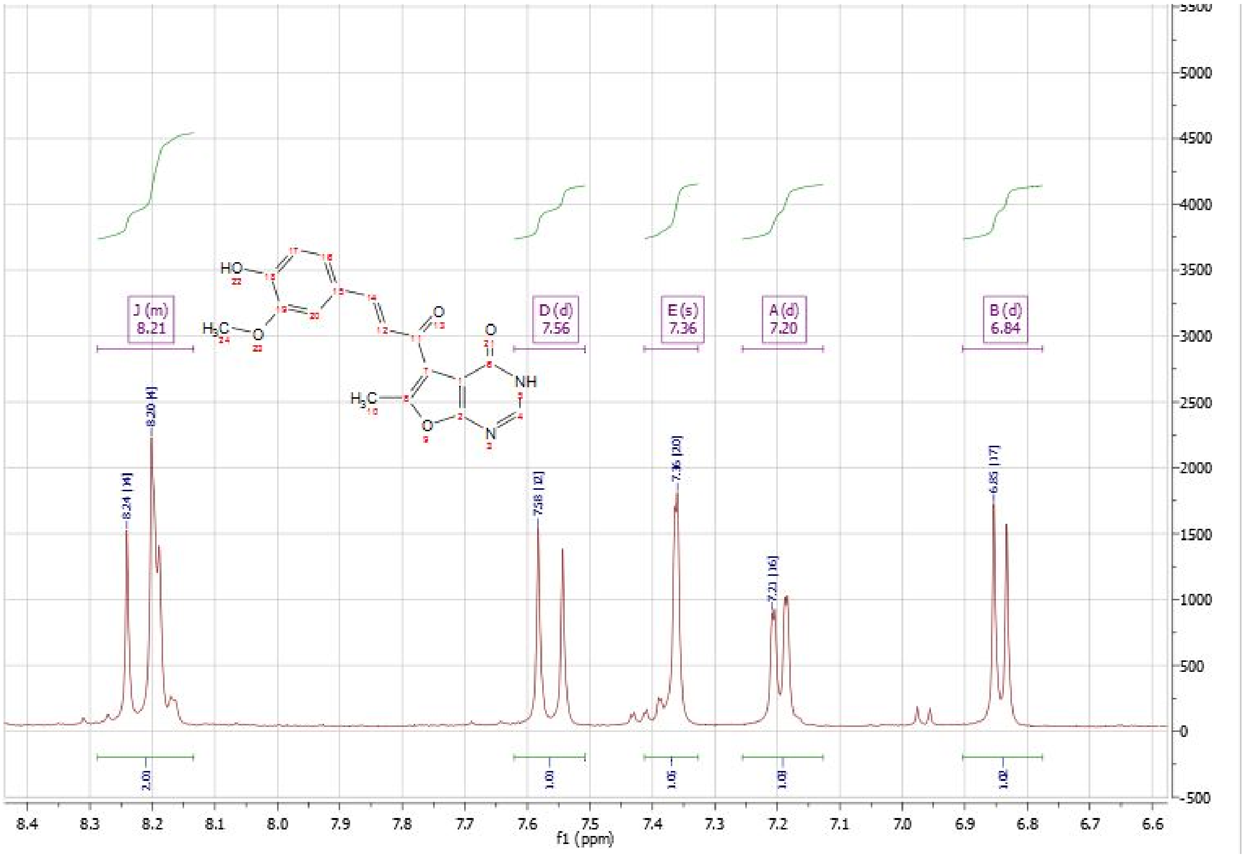

J (m)
8.21
D (d)
7.56
E (s)
7.36
A (d)
7.20
B (d)
6.84
HO
NH
N
O
2.01
1.00
1.05
1.03
1.02
f1 (ppm)

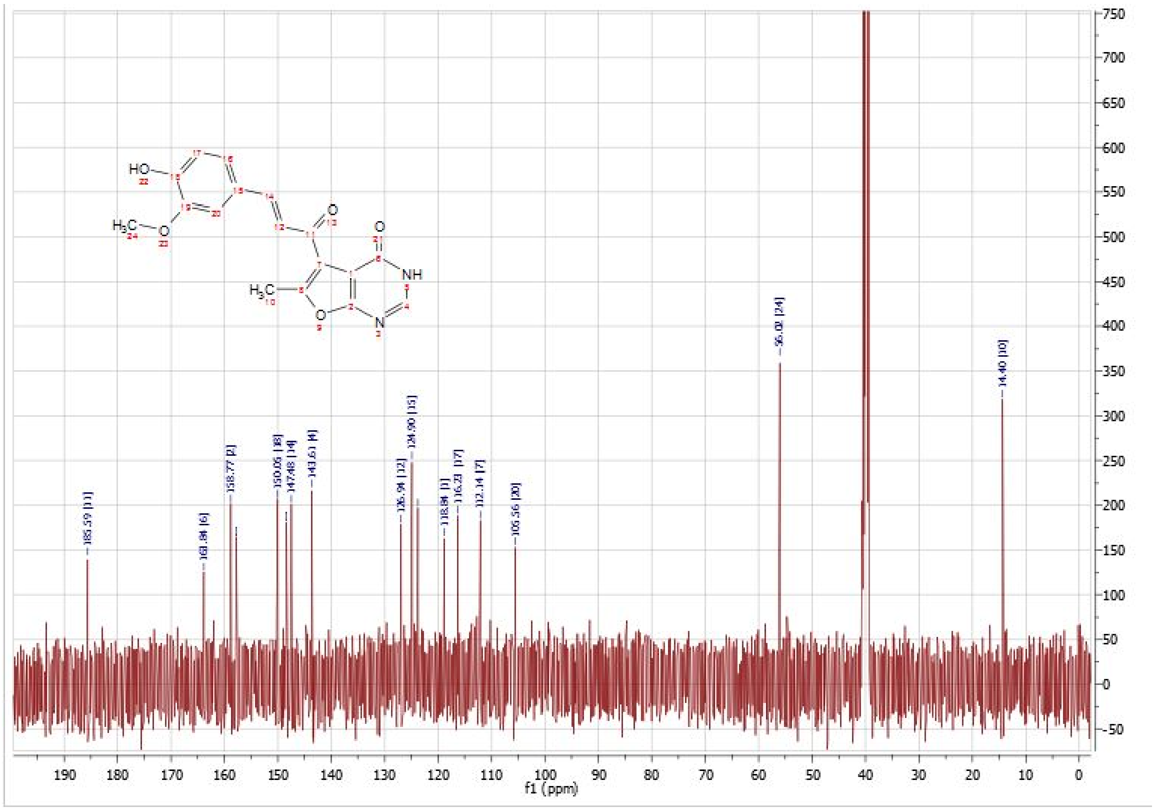

HO
NH
N
O
185.59
163.86
158.77
150.06
147.48
143.63
126.94
124.90
118.84
116.23
112.14
105.56
56.00
14.40
f1 (ppm)

(E)-5-(3-(3,4-dimethoxyphenyl)acryloyl)-6-methylfuro[2,3-*d*]pyrimidin-4(3H)-one (5j)

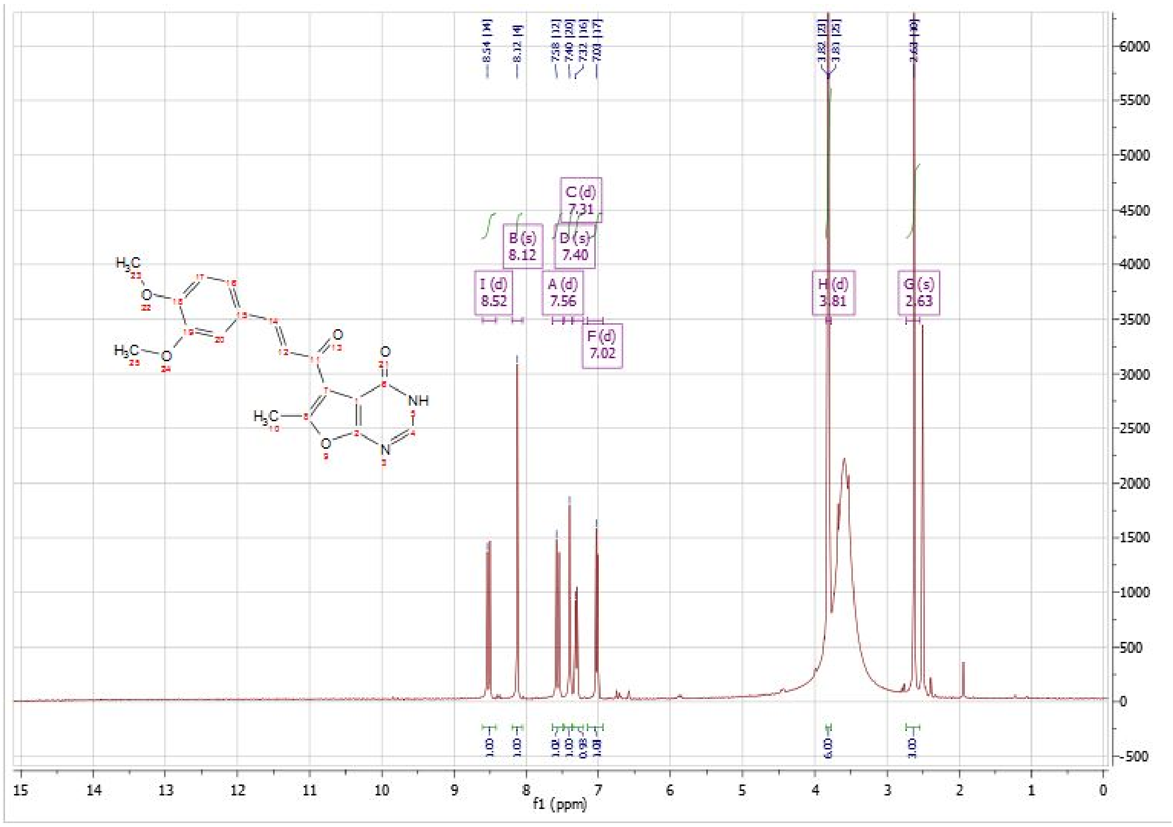

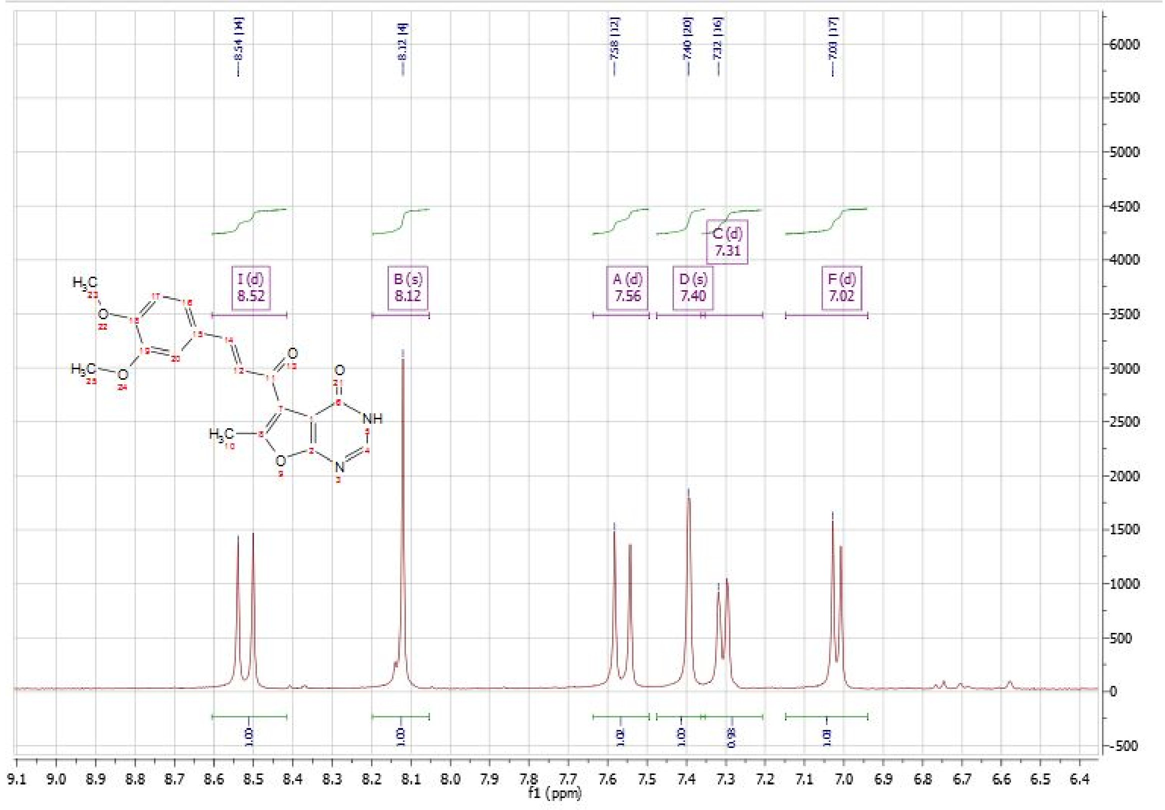

(E)-6-methyl-5-(3-(3,4,5-trimethoxyphenyl)acryloyl)furo[2,3-*d*]pyrimidin-4(3H)-one (5k)

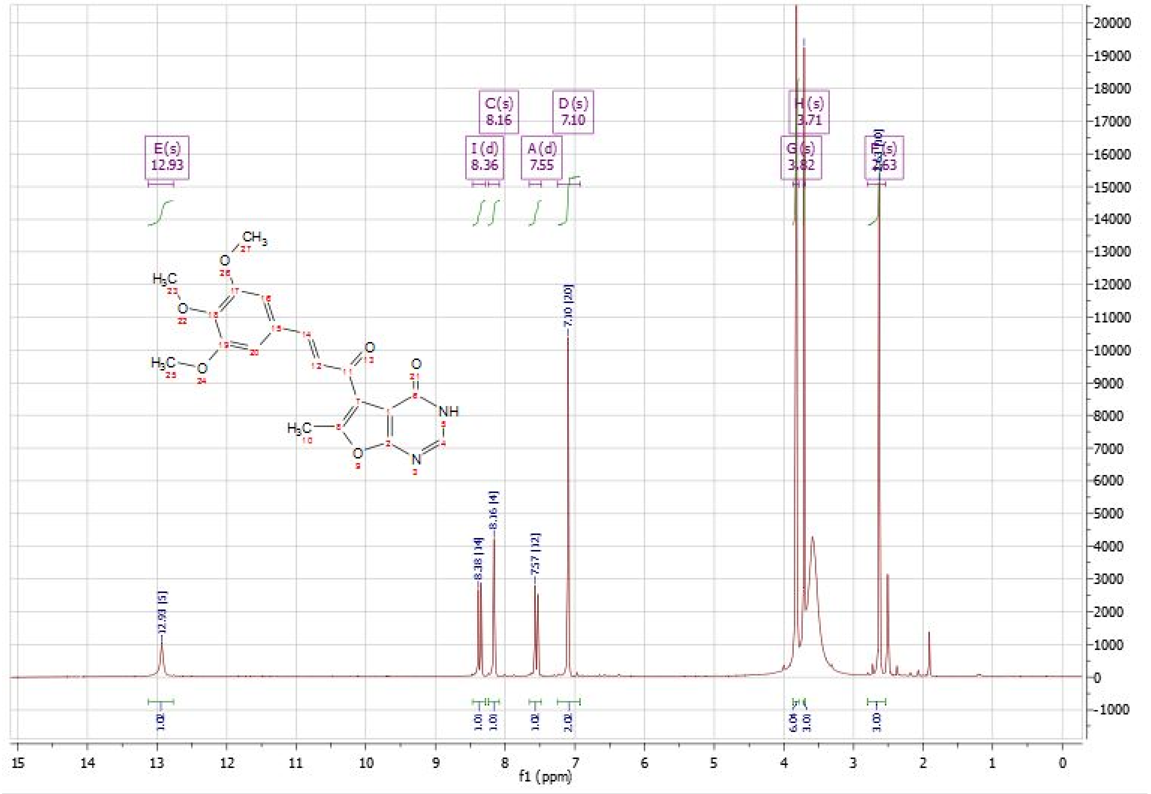

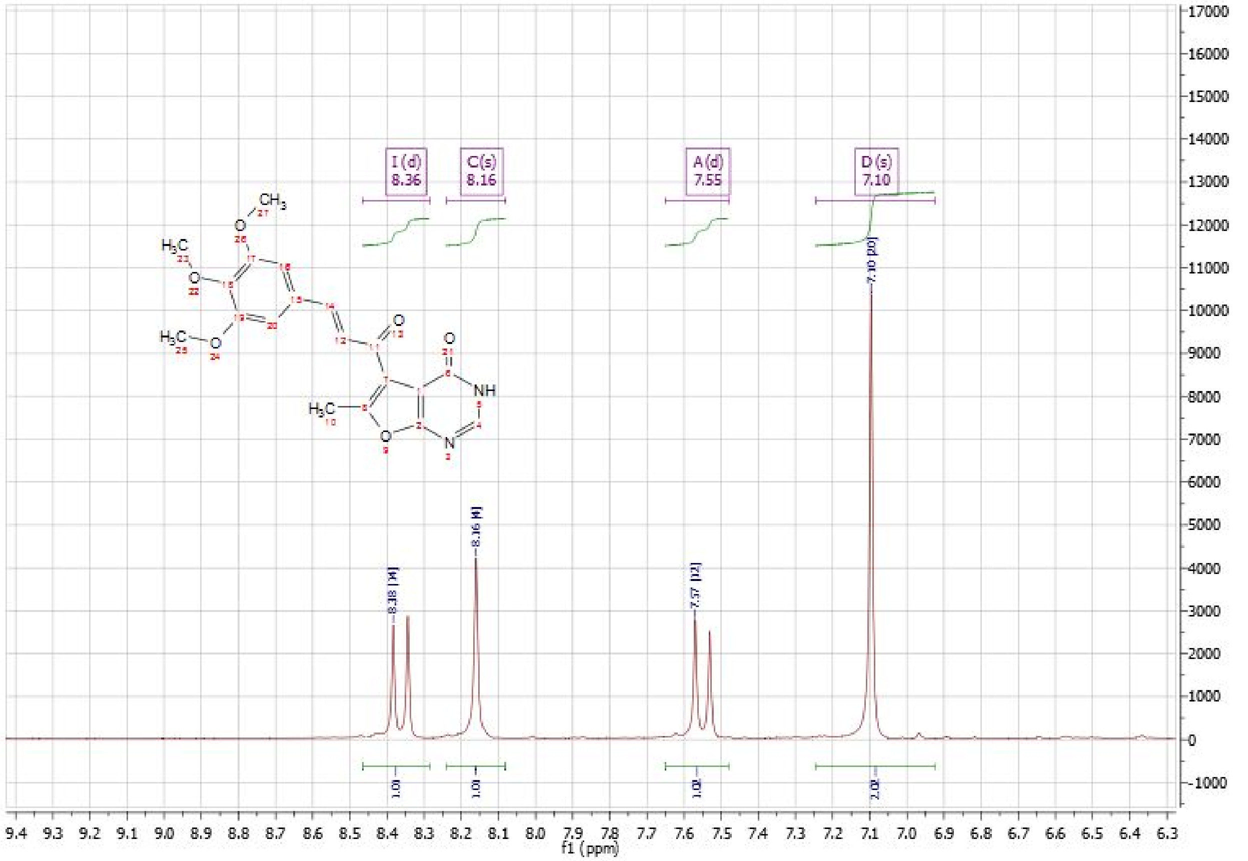

I (d)
8.36
C(s)
8.16
A(d)
7.55
D (s)
7.10
8.38 [M]
8.16 [M]
7.57 [I2]
7.10 [D]
1.01
1.01
1.02
2.03
f1 (ppm)
